# Dual regulation of TRPV1 channels by phosphatidylinositol *via* functionally distinct binding sites

**DOI:** 10.1016/j.jbc.2021.100573

**Published:** 2021-03-23

**Authors:** Aysenur Torun Yazici, Eleonora Gianti, Marina A. Kasimova, Bo-Hyun Lee, Vincenzo Carnevale, Tibor Rohacs

**Affiliations:** 1 Department of Pharmacology, Physiology and Neuroscience, Rutgers, New Jersey Medical School, Newark, New Jersey, USA; 2 Institute for Computational Molecular Science, Temple University, Philadelphia, Pennsylvania, USA

**Keywords:** phosphoinositides, TRP channel, TRPV1, ion channel, molecular dynamics, diC_8_, dioctanoyl, MD, molecular dynamics, PLC, phospholipase C, PtdIns, phosphatidylinositol, PtdIns(4,5)P_2_, phosphatidylinositol 4,5-bisphosphate, TRP, transient receptor potential, TRPV1, transient receptor potential vanilloid 1, TIRF, total internal reflection fluorescence

## Abstract

Regulation of the heat- and capsaicin-activated transient receptor potential vanilloid 1 (TRPV1) channel by phosphoinositides is complex and controversial. In the most recent TRPV1 cryo-EM structure, endogenous phosphatidylinositol (PtdIns) was detected in the vanilloid binding site, and phosphoinositides were proposed to act as competitive vanilloid antagonists. This model is difficult to reconcile with phosphatidylinositol 4,5-bisphosphate [PtdIns(4,5)P_2_] being a well-established positive regulator of TRPV1. Here we show that in the presence of PtdIns(4,5)P_2_ in excised patches, PtdIns, but not PtdIns(4)P, partially inhibited TRPV1 activity at low, but not at high capsaicin concentrations. This is consistent with PtdIns acting as a competitive vanilloid antagonist. However, in the absence of PtdIns(4,5)P_2_, PtdIns partially stimulated TRPV1 activity. We computationally identified residues, which are in contact with PtdIns, but not with capsaicin in the vanilloid binding site. The I703A mutant of TRPV1 showed increased sensitivity to capsaicin, as expected when removing the effect of an endogenous competitive antagonist. I703A was not inhibited by PtdIns in the presence of PtdIns(4,5)P_2_, but it was still activated by PtdIns in the absence of PtdIns(4,5)P_2_ indicating that inhibition, but not activation by PtdIns proceeds *via* the vanilloid binding site. In molecular dynamics simulations, PtdIns was more stable than PtdIns(4,5)P_2_ in this inhibitory site, whereas PtdIns(4,5)P_2_ was more stable than PtdIns in a previously identified, nonoverlapping, putative activating binding site. Our data indicate that phosphoinositides regulate channel activity *via* functionally distinct binding sites, which may explain some of the complexities of the effects of these lipids on TRPV1.

Transient receptor potential vanilloid 1 (TRPV1) is a heat- and capsaicin-activated Ca^2+^ permeable nonselective cation channel expressed in primary sensory neurons of the dorsal root ganglia and trigeminal ganglia (1). Stimulation of cell surface receptors by proinflammatory mediators sensitizes this channel to heat and capsaicin, a phenomenon thought to underlie the increased sensitivity to heat in inflammation. Accordingly, genetic deletion of this channel in mice essentially eliminated thermal hyperalgesia (2).

Phosphoinositides, especially phosphatidylinositol 4,5-bisphosphate [PtdIns(4,5)P_2_], are general regulators of many ion channels (3), including members of the transient receptor potential (TRP) family (4). In most cases, including the majority of TRP channels, PtdIns(4,5)P_2_ acts as a positive regulator, or a necessary cofactor for channel activity. Negative regulatory effects of this lipid have also been proposed for some TRP channels, for example TRPV3 (5). In some cases, negative and positive effects may coexist on the same channel for example on TRPC5 (6); for review and other examples see reference (4).

In resting conditions, PtdIns(4,5)P_2_ constitutes ∼1% of the plasma membrane phospholipids and serves as a substrate for phospholipase C (PLC) enzymes (7). Its immediate precursor PtdIns(4)P is found at similar concentrations, and it can also be hydrolyzed by PLC, even though less efficiently than PtdIns(4,5)P_2_. Most research on ion channel regulation focused on PtdIns(4,5)P_2_ (3), but distinct roles for PtdIns(4)P have also been proposed (8, 9). PtdIns is the precursor of PtdIns(4,5)P_2_ and PtdIns(4)P, and very little is known about its effect on ion channel function.

Phosphoinositides modulate TRPV1 activity in a complex manner; both positive and negative effects of these lipids have been proposed (10). It was first suggested that PtdIns(4,5)P_2_ negatively regulates TRPV1 and that relief from this inhibition plays a major role in sensitization of the channel upon PLC activation by proinflammatory receptor stimulation (11).

Subsequent studies from different laboratories however unanimously demonstrated that PtdIns(4,5)P_2_, or its precursor PtdIns(4)P, applied directly to excised inside out patches potentiates TRPV1 activity (12, 13, 14, 15, 16). MgATP also potentiated channel activity in excised inside out patches *via* stimulated synthesis of endogenous PtdIns(4,5)P_2_ (15). When the purified channel was incorporated into planar lipid bilayers, the capsaicin- and heat-induced activity of TRPV1 also depended on the presence of PtdIns(4,5)P_2_, or PtdIns(4)P, demonstrating a direct effect on the channel (15, 17).

In intact cells, channel activity induced by capsaicin or low pH requires the presence of PtdIns(4,5)P_2_ and/or its immediate precursor PtdIns(4)P. Depletion of these lipids by specific inducible phosphoinositide phosphatases resulted in diminished channel activity (8, 9, 14, 18). PtdIns(4,5)P_2_ and PtdIns(4)P are also depleted by Ca^2+^-induced activation of PLC when TRPV1 is activated by saturating capsaicin concentrations, and this effect plays an important role in desensitization of TRPV1 (9, 13, 18, 19). These data provided overwhelming evidence for PtdIns(4,5)P_2_ being a positive cofactor/regulator of TRPV1, see more detailed discussion in (10).

The most recent and highest resolution cryo-EM structure of TRPV1 revealed the presence of an endogenous phosphoinositide partially occupying the vanilloid binding site. The lipid was modeled as PtdIns, but it was argued that this site can accommodate a range of phosphoinositide species, and it was proposed that phosphoinositides act as competitive vanilloid antagonist (20). However, this model is difficult to reconcile with the strong evidence for PtdIns(4,5)P_2_ and PtdIns(4)P being positive regulators of TRPV1 in cellular membranes.

Most TRPV channels are positively regulated by PtdIns(4,5)P_2_ (4). The only TRPV channel where the location of the PtdIns(4,5)P_2_ binding site has been determined by cryo-EM is TRPV5. The PtdIns(4,5)P_2_-bound structure of TRPV5 shows an open channel (21), which is consistent with this lipid serving an obligate cofactor for TRPV5 (22, 23). A binding site responsible for the activating effect of PtdIns(4,5)P_2_ in TRPV1 involving the S4-S5 linker and the proximal C-terminal TRP-domain was predicted based on the binding modes by molecular docking of the lipid against the cryo-EM structure of TRPV1 (16). The location of this binding site is also supported by experimental data showing lower apparent affinity for PtdIns(4,5)P_2_ activation in excised patches in the K694A, R575A, and R579A mutants (16). This putative binding site in TRPV1 is very similar to the location of PtdIns(4,5)P_2_ in the cryo-EM structure of TRPV5 (21).

Here, we show that in excised inside out patches PtdIns, but not PtdIns(4)P, partially inhibited channel activity in the presence of PtdIns(4,5)P_2_. The inhibitory effect showed an inverse correlation with the concentration of capsaicin, and mutating residues predicted to interact with PtdIns, but not with capsaicin, resulted in higher sensitivity to capsaicin activation and reduced inhibition by PtdIns. These data are compatible with PtdIns acting as a competitive vanilloid antagonist. In the absence of PtdIns(4,5)P_2_, on the other hand, PtdIns partially stimulated TRPV1 activity especially in the presence of high capsaicin concentrations, an indication that PtdIns also binds to an activating lipid binding site. We used molecular modeling to characterize the binding of phosphoinositides to both the site partially overlapping with the vanilloid binding site (inhibitory site) and the one located between the S4-S5 linker and the proximal C-terminal TRP-domain (activating site). Theoretical structural models are consistent with the hypothesis that phosphoinositides act primarily on these nonoverlapping binding sites, one with activating and the other with inhibiting effect.

## Results

To study the effects of PtdIns and PtdIns(4,5)P_2_ on capsaicin-induced TRPV1 activity, we expressed the channel in *Xenopus* oocytes and performed excised inside out patch clamp and two electrode voltage clamp experiments to measure TRPV1 currents. We complemented the experimental work with computational modeling, *i.e*., binding site mapping, molecular docking and molecular dynamics (MD) simulations.

### PtdIns partially inhibits TRPV1 in excised patches in the presence of PtdIns(4,5)P_2_

**Figure 1:**
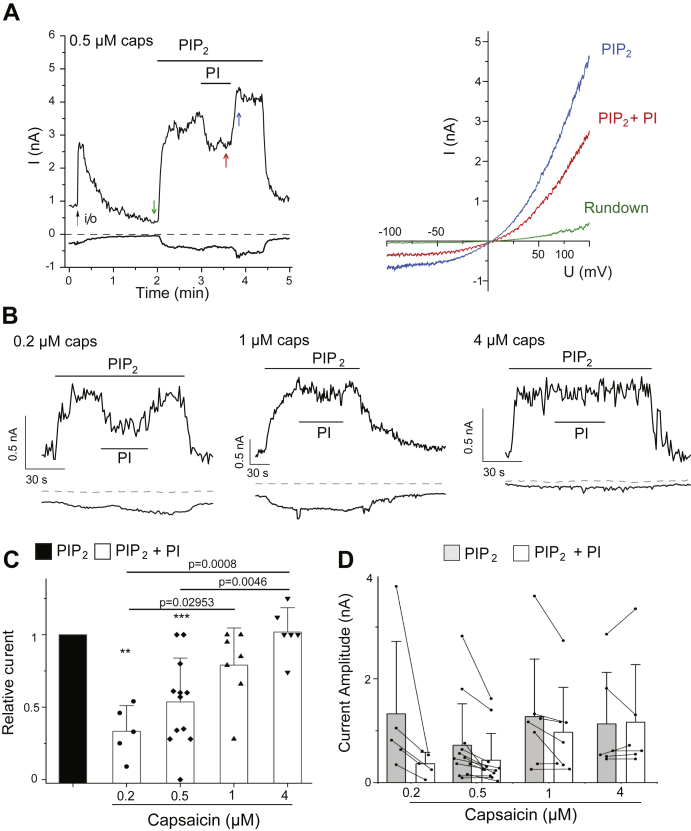
**Phosphatidylinositol inhibits TRPV1 in excised patches at a low but not at high capsaicin concentrations in the presence of PtdIns(4,5)P**_**2**_. Excised inside out patch clamp experiments were performed as described in the Experimental procedures section in *Xenopus* oocytes injected with the TRPV1 cRNA. *A*, *left panel*, representative current traces at −100 and 100 mV in the presence of 0.5 μM capsaicin in the patch pipette. The establishment of the inside out configuration is marked with an *arrow*, and the applications of 50 μM diC_8_ PtdIns(4,5)P_2_ (PIP_2_) and 50 μM diC_8_ PtdIns (PI) are shown by the horizontal lines. *Right panel*, individual ramp current traces at the time points indicated by the colored *arrows* from the *left panel*. *B*, representative current traces at −100 and 100 mV, in the presence of 0.2 μM (*left*), 1 μM (*middle*), and 4 μM (*right*) capsaicin in the patch pipette, and the applications of 50 μM diC_8_ PtdIns(4,5)P_2_ and 50 μM diC_8_ PtdIns are shown by the *horizontal lines*. *C*, summary of the data normalized to the currents evoked by PtdIns(4,5)P_2_ mean ± SD and scatter plots. Statistical significance between the effects of PtdIns at different capsaicin concentrations was calculated with one-way ANOVA, and *p* values are shown for Bonferroni post hoc test. Asterisks above the individual columns show significance for PtdIns inhibition (difference from 1) calculated with one sample *t* test (∗∗*p* = 0.00109, ∗∗∗*p* = 0.00025). The correlation coefficient between the concentration of capsaicin and the relative current during PtdIns application was also calculated and showed a Pearson’s coefficient of 0.618 and *p* = 0.000276. *D*, summary of raw current amplitudes from the same experiments, mean ± SD and scatter plots; data points from the same patches are connected. diC8, dioctanoyl; PtdIns, phosphatidylinositol; PtdIns(4,5)P_2_, phosphatidylinositol 4,5-bisphosphate; TRPV1, transient receptor potential vanilloid 1.

To test if PtdIns inhibits TRPV1 activity, we performed excised inside out patch clamp experiments where we can directly apply phosphoinositides to the cytoplasmic leaflet of the plasma membrane. We found that TRPV1 activity shows a decrease (rundown) after patch excision in the presence of 0.5 μM capsaicin in the patch pipette (Fig. 1*A*), which is consistent with our earlier findings (15). This rundown is a characteristic of PtdIns(4,5)P_2_ dependent ion channels caused by dephosphorylation of PtdIns(4,5)P_2_ by phosphatase enzymes in the patch membrane. When we applied the water soluble dioctanoyl (diC_8_) PtdIns(4,5)P_2_ (50 μM), channel activity was restored. Co-application of 50 μM diC_8_ PtdIns induced a partial, reversible inhibition of TRPV1 activity (Fig. 1*A*). The effect of PtdIns, was on average ∼50%, and while statistically significant, it was highly variable, ranging from no inhibition to full inhibition (Fig. 1, *C* and *D*). Next, we tested if the inhibitory effect of PtdIns is decreased at higher and increased at lower capsaicin concentrations, as expected if the lipid acts as a competitive vanilloid antagonist. Figure 1, *B*–*D* shows that the inhibitory effect of PtdIns showed an inverse correlation with the concentration of capsaicin, showing essentially no inhibition at 4 μM capsaicin and ∼70% inhibition at 0.2 μM capsaicin. We also tested additional concentrations of PtdIns. We found that 25 μM PtdIns was less effective than 50 μM in the presence of 0.2 μM capsaicin, and 100 μM PtdIns was similarly effective to 50 μM both in the presence of 0.2 μM and 1 μM capsaicin (Fig. S1).

**Figure 2:**
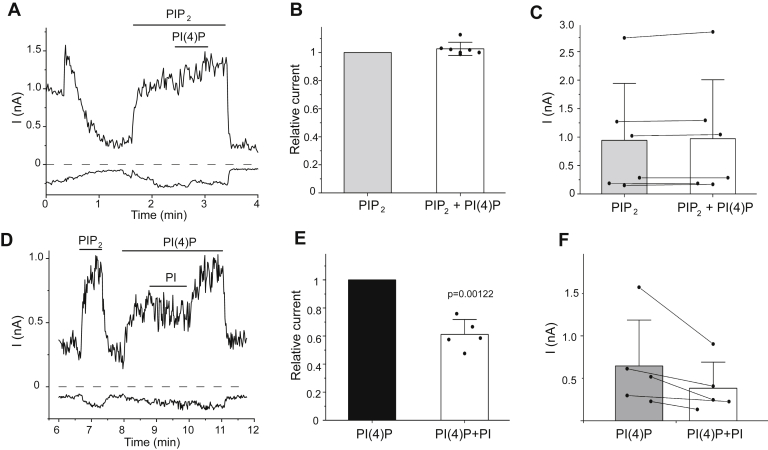
**PtdIns(4)P does not inhibit TRPV1, but PtdIns inhibits currents evoked by PtdIns(4)P**. Excised inside out patch clamp experiments were performed as shown in Figure 1 with 0.5 μM capsaicin in the patch pipette. *A*, representative trace shown at −100 and 100 mV; the applications of 50 μM PtdIns(4,5)P_2_ and 50 μM PtdIns(4)P are indicated by the *horizontal lines*. *Dashed line* shows zero current. *Arrow* indicates the establishment of the inside out configuration. *B*, summary of the data normalized to the effect of PtdIns(4,5)P_2_ at 100 mV. To compensate for the spontaneous increase of currents, we divided the mean current amplitude during the application of PtdIns(4)P with the average of current amplitudes in the 20 s periods before and after the application of PtdIns(4)P. *C*, summary of raw current amplitudes from the same experiments; data points from the same patches are connected. Data are shown as mean ± SD and scatter plots. *D*, representative trace shown after current rundown at −100 and 100 mV; the applications of PtdIns(4,5)P_2_, 50 μM PtdIns(4)P, and 50 μM PtdIns are indicated by the *horizontal lines*. *E*, summary of the data normalized to the effect of PtdIns(4)P; data analysis was performed similar to that in *panel B*. Statistical significance was calculated with one sample *t* test (difference from 1). *F*, summary of raw current amplitudes from the same experiments, data points from the same patches are connected. Data are shown as mean ± SD and scatter plots. PtdIns, phosphatidylinositol; PtdIns(4,5)P_2_, phosphatidylinositol 4,5-bisphosphate; TRPV1, transient receptor potential vanilloid 1.

PtdIns(4)P is the precursor of PtdIns(4,5)P_2_. This lipid was shown to also increase channel activity, but with a lower apparent affinity than PtdIns(4,5)P_2_ (13, 14). Unlike PtdIns, 50 μM PtdIns(4)P did not inhibit channel activity in the presence of 50 μM PtdIns(4,5)P_2_ and 0.5 μM capsaicin (Fig. 2, *A*–*C*). PtdIns (50 μM) on the other hand inhibited TRPV1 when the channel was activated by 50 μM PtdIns(4)P in the presence of 0.5 μM capsaicin (Fig. 2, *D*–*F*).

### PtdIns partially activates TRPV1 in the absence of PtdIns(4,5)P_2_ at high capsaicin concentrations

**Figure 3:**
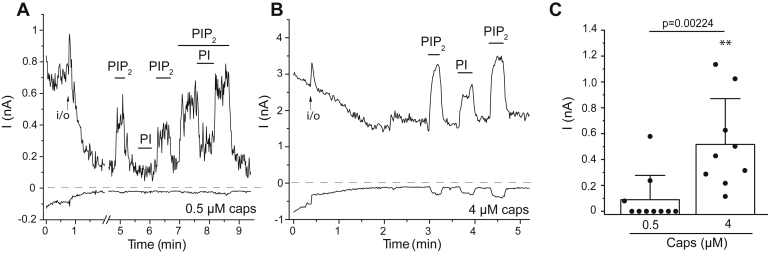
**PtdIns partially activates TRPV1 in the presence of high capsaicin concentrations.** Excised inside out patch experiments were performed as described in the Experimental procedures section in *Xenopus* oocytes injected with the TRPV1 cRNA. *A* and *B*, representative current traces at −100 and 100 mV, in the presence of 0.5 μM (*A*) and 4 μM (*B*) capsaicin in the patch pipette; the establishment of the inside out configuration is marked with an *arrow*, and the applications of 25 μM diC_8_ PtdIns(4,5)P_2_ and 100 μM diC_8_ PtdIns are indicated by the *horizontal lines*. *Dashed lines* show zero current. For the co-application of PIP_2_ and PI in *panel A*, the concentration of diC_8_ PtdIns(4,5)P_2_ was 50 μM. *C*, summary of the current amplitudes evoked by 100 μM PtdIns, mean ± SD and scatter plots. Statistical significance of the difference between the current amplitudes evoked at 0.5 μM and 4 μM capsaicin was calculated by the Mann-Whitney test. The effect of PtdIns at 0.5 μM capsaicin was not significantly different from 0 with the one sample Wilcoxon Signed Rank test (*p* = 0.18). The effect of PtdIns at 4 μM capsaicin was significantly different form 0 with one sample *t* test (∗∗*p* = 0.00236). diC8, dioctanoyl; PtdIns, phosphatidylinositol; PtdIns(4,5)P_2_, phosphatidylinositol 4,5-bisphosphate; TRPV1, transient receptor potential vanilloid 1.

Our data support the idea that PtdIns is a competitive channel inhibitor at the binding site overlapping with that for vanilloids. We found earlier that after channel rundown, very high concentrations (500 μM) of long acyl chain (AASt) PtdIns partially reactivated TRPV1 at 0.5 μM capsaicin (15). Here, we revisited this and tested the effect of diC_8_ PtdIns at different capsaicin concentrations in the absence of PtdIns(4,5)P_2_. We found that 100 μM diC_8_ PtdIns had no effect in the majority of patches in the presence of 0.5 μM capsaicin (Fig. 3*A*), even though in some patches (three out of 10) it induced a small, partial activation, see Figure 3*C* for summary.

In the presence of 4 μM capsaicin on the other hand, 100 μM diC_8_ PtdIns reliably reactivated TRPV1 (Fig. 3, *B* and *C*). The amplitudes of the PtdIns-induced currents were 60.4 ± 11.2% (SD) of that induced by 25 μM diC_8_ PtdIns(4,5)P_2_ in the same patches. These data indicate that capsaicin not only increases the apparent affinity for PtdIns(4,5)P_2_ in activating TRPV1 (13) but also decreases the selectivity of the activating phosphoinositide binding site allowing PtdIns to partially activate the channel. An alternative possibility is that in the presence of low capsaicin concentrations, PtdIns binds to both the activating and inhibitory sites, and the two effects largely cancel each other, whereas at high capsaicin, PtdIns is displaced from the inhibitory site, unmasking an activating effect.

### Location of the inhibitory and the putative activating phosphoinositide binding sites in TRPV1

Our data show that PtdIns partially inhibits TRPV1 in the presence of PtdIns(4,5)P_2_, and this effect is more prevalent at lower capsaicin concentrations, which is compatible with PtdIns competing with capsaicin for an overlapping binding site (*i.e.,* the inhibiting site). We also found however that PtdIns partially activates TRPV1 in the absence of PtdIns(4,5)P_2_, and this effect is more prevalent at higher capsaicin concentrations. These two findings are hard to reconcile with PtdIns binding to a single binding site. We therefore hypothesize that at high capsaicin concentrations, PtdIns activates the channel through a distinct binding site, presumably the same where PtdIns(4,5)P_2_ binds to activate the channel (*i.e.,* the activating site).

**Figure 4:**
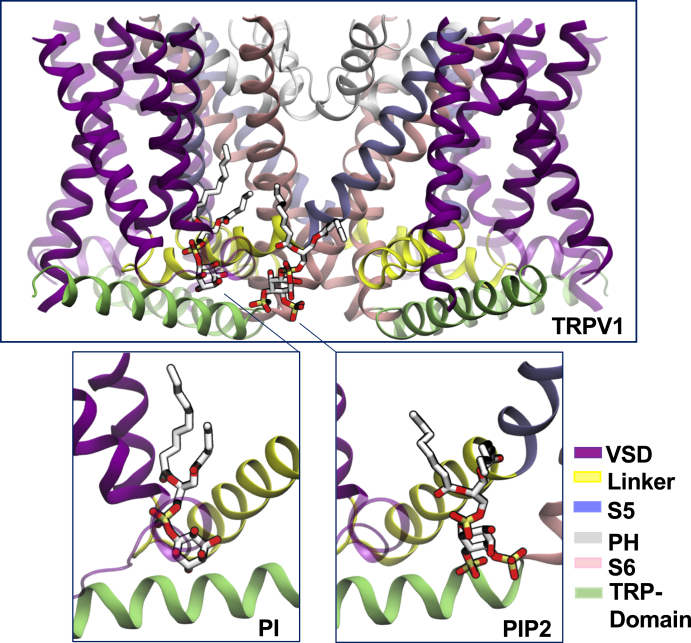
**Location of the inhibitory and the putative activating phosphoinositide binding sites in TRPV1 (*Top panel*)**. The transmembrane portion of the TRPV1 channel is shown (residues 430–713; side view), with phosphoinositides bound at the inhibiting (*left*) and activating (*right*) sites. The inhibiting site corresponds to the location of the phosphoinositide solved by Cryo-EM (20), and partially overlaps (but doesn’t fully coincide) with the vanilloid pocket; the activating site matches the experimentally observed location for the binding of PtdIns(4,5)P_2_ to TRPV5 (21). The inhibiting and activating binding sites are shown with PtdIns (PI) and PtdIns(4,5)P_2_, (PIP_2_) respectively. The binding sites are shown for one subunit only using a different color scheme for each domain of the channel: the S1-S4-helix bundle is *purple* (residues 430–556), the S4-S5 linker is *yellow* (residues 557–575), S5 is *ice-blue* (residues 576–598), the pore helix (PH) is *white* (residues 599–655), S6 is *pink* (residues 656–689), and TRP domain is *green* (residues 690–713). In the *insets*, phosphoinositides are shown as *balls* and *sticks*, atoms color coded by element: C, O, and P atoms are *gray*, *red*, and *yellow*, respectively; hydrogen atoms not shown. PtdIns, phosphatidylinositol; PtdIns(4,5)P_2_, phosphatidylinositol 4,5-bisphosphate; TRPV1, transient receptor potential vanilloid 1.

To assess the feasibility of this two binding site model, we compared the PtdIns/vanilloid binding site (20) and the putative PtdIns(4,5)P_2_ binding site, which was proposed to be responsible for the activating effect of PtdIns(4,5)P_2_ by the Latorre group (16). This binding site was identified by computational docking of PtdIns(4,5)P_2_ to the cryo-EM structure of TRPV1 (24) and was experimentally supported by showing reduced PtdIns(4,5)P_2_ activation of the R575A, R579A, and K694A mutants (16). This binding site features a strikingly similar location to the PtdIns(4,5)_2_ binding site recently identified in the cryo-EM structure of the related TRPV5 channel (21). The TRPV5 structure was determined with diC_8_ PtdIns(4,5)P_2_ added to the purified protein, and the lipid induced a clear conformational change indicating channel opening (21). While other potential PtdIns(4,5)P_2_ binding sites in TRPV1 were also proposed (10, 25, 26), given the concordance of structural data on TRPV5 (21) and computational and functional data on TRPV1 (16), here we focus on this putative binding site to explain the complex effects of phosphoinositides on TRPV1. First, we superimposed the structures of TRPV1 (20) and TRPV5 (21) to evaluate whether or not PtdIns and PtdIns(4,5)P_2_ binds to the same region of the channel. Comparison between the two structures clearly shows that the two phosphoinositide binding sites, namely the “inhibiting site” and the “activating site”, are distinct, adjacent yet nonoverlapping (Figs. 4 and S2).

### Functional effects of mutations predicted to affect PtdIns but not capsaicin binding

**Figure 5:**
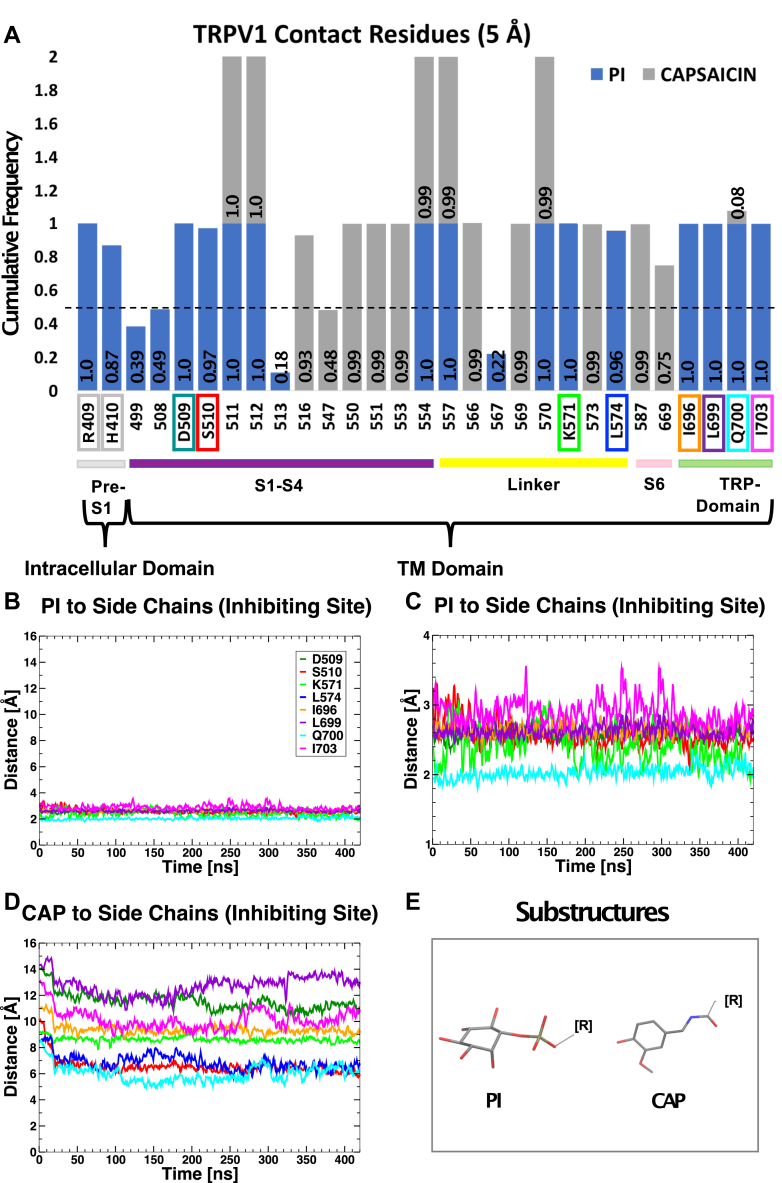
**Residues in contact with PtdIns but not with capsaicin**. *A***,** analyses of residue contacts (from MD) identify what residues interact with PtdIns (PI) or with capsaicin. Not all the sidechains are in contact with both PtdIns and capsaicin, suggesting that the two binding sites (PtdIns and capsaicin) are not identical. For each amino acid residue, frequency distributions of contacts, with either PtdIns, capsaicin, or both, are shown as stacked bar-charts in different colors. Frequencies of individual ligands are added (summed over PtdIns and capsaicin, for a cumulative maximum value of 2) to generate cumulative frequencies of contacts, established by each amino acid residue. *Dotted line* indicates 0.5 frequency values (contacts established at least in 50% of simulation frames). *B–E*, minimum distances between PtdIns (*panels* B and *C*) or capsaicin (*panel D*) and the sidechains shown in *panel A*. Distances are calculated between amino acid sidechain atoms and lipid head groups (substructures in *panel E*) and averaged over all subunits. *C*, close-up of the time series shown in *panel B*. MD, molecular dynamics; PtdIns, phosphatidylinositol.

Next, we computationally identified residues, which are in contact with PtdIns, but not with capsaicin in the vanilloid binding site (5 Å cutoff): R409 and H410 in pre-S1, D509 and S510 in the S2–S3 loop, K571 and L574 in the S4–S5 segment, and I696, L699, Q700, and I703 in the TRP box (Fig. 5*A*).

In principle, mutation of a residue important for PtdIns binding, but not for capsaicin binding, should reduce inhibition of the channel by endogenous PtdIns, and it would be predicted to increase sensitivity to activation by capsaicin (left shifted concentration response curve), because of weakening inhibition by an endogenous competitive inhibitor. We noticed that R409 and H410 are solvent exposed (Fig. S3) and, on average, more than 4 Å farther away from PtdIns, therefore not in direct contact with it. Mutation of these residues is thus unlikely to produce large changes in PtdIns affinity. We thus focused our attention to D509, S510, K571, L574, I696, L699, Q700, and I703. All these sidechains are in close contact with PtdIns (with minimum distances smaller than 3 Å, Fig. 5, *B* and *C*) yet are seemingly not interacting with capsaicin (minimum distances larger than 6 Å, Fig. 5*D*).

**Figure 6:**
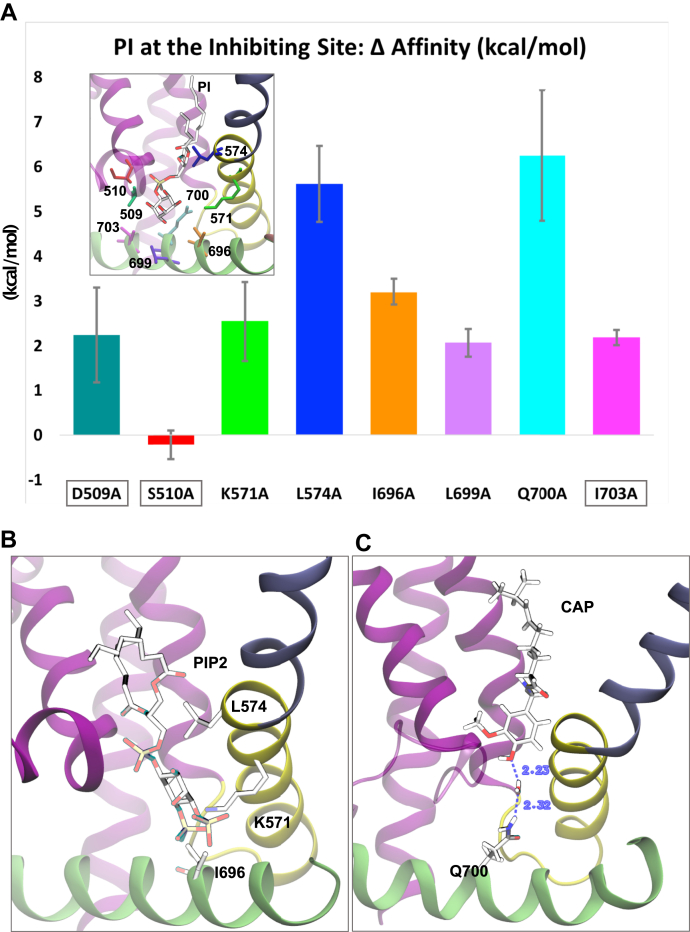
**Change in affinity upon mutation of inhibiting binding site residues.***A*, the histogram plot shows the ΔΔG (kcal/mol) or change in PtdIns binding free energy, upon mutation into alanine of the binding site residues. Values are averaged over individual subunits and multiple configurations sampled from the MD trajectory. Error bars show the standard deviation. *Inset*, binding mode of PtdIns (PI) at the inhibiting site. Residues selective for PtdIns binding and subject to computational mutation analysis are shown as stick representation and colored to match the histogram. *B*, stick representation of the sidechains of L574, K571, and I696 and of PtdIns(4,5)P_2_ (PIP_2_) in the activating site (note the close proximity). *C*, sidechain of residue Q700 observed in our molecular dynamics simulations to interact with capsaicin through an intervening hydrogen-bonded water molecule, see also Figure S4. In (*B*) and (*C*), atom coloring follows the standard CPK rules. MD, molecular dynamics; PtdIns, phosphatidylinositol; PtdIns(4,5)P_2_, phosphatidylinositol 4,5-bisphosphate.

We thus considered these contacts as *bona fide* interactions and proceeded with a more computationally intensive characterization of these sidechains. In particular, we calculated the change in PtdIns affinity (ΔΔG) upon mutation of each sidechain into alanine. Figure 6 shows that, with the exception of S510A, all the alanine mutants have a large destabilizing effect on PtdIns binding. Before proceeding with experimental testing of these mutants, we re-examined in detail the interactions between these sidechains and all the ligands involved (PtdIns(4,5)P_2_, PtdIns, and capsaicin). We noticed that I696, K571, and L574 also form tight interactions with PtdIns(4,5)P_2_ in the putative activating site (Fig. 6*B*), and therefore, their mutations are likely to result in complex phenotypes. The same is true for Q700, which is involved in an indirect interaction with capsaicin through an intervening hydrogen-bonded water molecule observed in a large fraction of the simulation frames (Figs. 6*C* and S4). Accordingly, we found that the S510A, Q700A, and the L574A mutants had slightly right shifted capsaicin concentration dependence (Fig. S5*A*). Of the remaining list of amino acids, L699A had been reported to show a capsaicin sensitivity very similar to that of WT channels (27).

**Figure 7:**
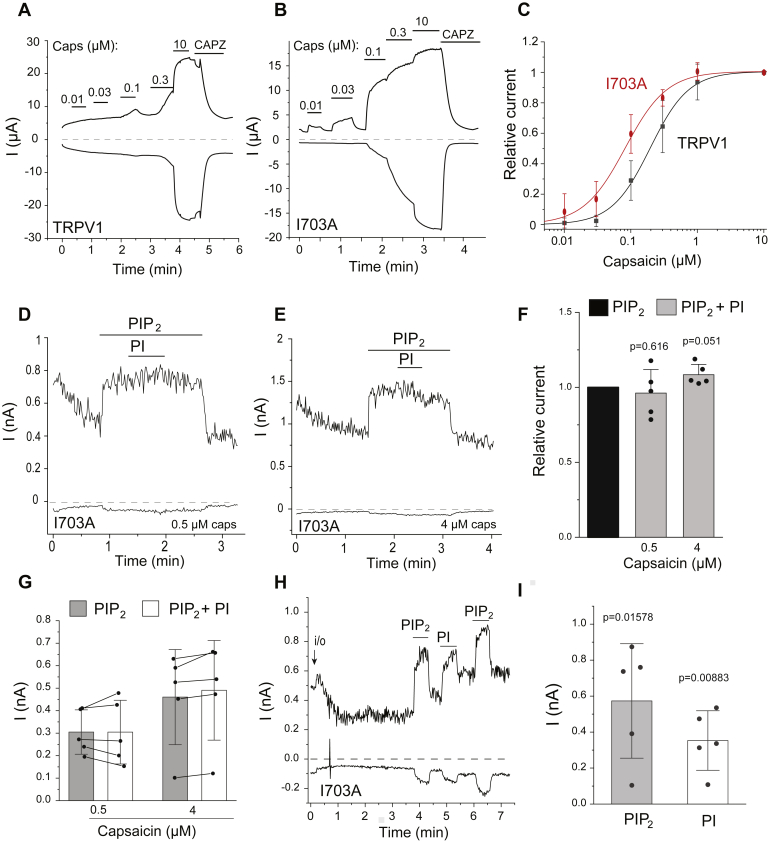
**The I703A mutant is more sensitive to activation by capsaicin and not inhibited by PtdIns.***A–C*, two electrode voltage clamp experiments were performed as described in the Experimental procedures section in *Xenopus* oocytes injected with the TRPV1 WT and I703A cRNA. *A* and *B*, representative current traces at −100 and +100 mV, the applications of different concentrations of capsaicin (μM), and 50 μM capsazepine (CAPZ) are indicated by the horizontal lines. *C*, hill plots for the concentration dependence of activation by capsaicin, mean ± SD, n=7 to 12. The EC_50_ for capsaicin activation was 0.196 ± 0.012 μM for WT and 0.083 ± 0.005 μM for I703A. *D* and *E*, excised inside out patch clamp experiments in *Xenopus* oocytes injected with the TRPV1-I703A cRNA. *D*, representative current trace at −100 and 100 mV in the presence of 0.5 μM capsaicin in the patch pipette; the applications of 50 μM diC_8_ PtdIns(4,5)P_2_ and 50 μM diC_8_ PtdIns are shown with the horizontal lines. *E*, similar measurement with 4 μM capsaicin in the patch pipette. *F*, summary of the data: mean ± SD and scatter plots; experiments also included same day control measurements with WT TRPV1 expressing oocytes; those data are included in the data summary in Figure 1*C*. Statistical significance (difference from 1) was calculated by one sample *t* test. *G*, summary of raw current amplitudes from the same experiments, and data points from the same patches are connected. *H*, representative excised inside out patch measurement showing activation of the I703A mutant by 100 μM diC_8_ PtdIns and by 25 μM diC_8_ PtdIns(4,5)P_2_. *I*, summary of the raw current amplitudes induced by PtdIns(4,5)P_2_ and PtdIns, mean ± SD and scatter plots. Statistical significance (difference from 0) was calculated by one sample *t* test. diC8, dioctanoyl; PtdIns, phosphatidylinositol; PtdIns(4,5)P_2_, phosphatidylinositol 4,5-bisphosphate; TRPV1, transient receptor potential vanilloid 1.

For these reasons, we focused on residues D509 and I703. We found that both the I703A (Fig. 7, *A*–*C*) and D509A (Fig. S5*A*) mutants showed left-shifted concentration dependence for capsaicin activation. Maximal current amplitudes were similar to WT TRPV1 for both mutants (Fig. S6*A*). We also transfected HEK293 cells with GFP-tagged WT TRPV1 and GFP-tagged I703A mutant and performed total internal reflection fluorescence (TIRF) imaging experiments. In TIRF microscopy, the excitation light hits the glass–cell interface at a narrow critical angle, so that it is totally reflected. The generated evanescent wave penetrates the cell to less than 100 nm, predominantly exciting the plasma membrane. Both constructs showed punctate distribution in the TIRF mode, which is consistent with earlier reports (12). In the zero-angle mode (widefield), mutant and WT TRPV1 showed both plasma membrane and intracellular localization, which is consistent with the localization of TRPV1 not only in the plasma membrane but also in the endoplasmic reticulum (28, 29). While both TIRF fluorescence (plasma membrane) and total fluorescence (widefield) showed a small decrease in the I703 mutant, their ratio was very similar for the mutant and the WT channel, showing that plasma membrane trafficking was not altered by the mutation (Fig. S6, *B–F*).

We also found that the D509A and I703A mutants became more sensitive to activation by low pH (Fig. S7). This latter finding is not unexpected, as vanilloid antagonists such as capsazepine and iodinated resiniferatoxin inhibit TRPV1 responses not only to capsaicin but also to low pH (30).

Next, we tested if these mutations also altered inhibition of TRPV1 by PtdIns in excised patches. Figure 7, *D*–*F* shows that 50 μM PtdIns did not inhibit the I703A mutant in excised patches in the presence of 50 μM PtdIns(4,5)P_2_ and either 0.5 μM or 4 μM capsaicin. We also found that the I703A mutant was activated by 100 μM diC_8_ PtdIns in the presence of 4 μM capsaicin and absence of PtdIns(4,5)P_2_ (Fig. 7, *H* and *I*), indicating that the mutation did not affect the activating binding site.

The D509A mutant on the other hand was clearly inhibited by PtdIns (Fig. S5, *C* and *D*). We also found that the D509A mutant had reduced rectification compared with WT TRPV1 and the I703 mutant when activated by capsaicin (Fig. S5*E*) or by low pH (Fig. S7*E*), especially at low stimulation strengths. D509A also had significantly slower deactivation compared with WT and I703 after removal of capsaicin combined with application of the TRPV1 antagonist capsazepine (Fig. 7, *A* and *B* and Fig. S5, *B* and *F*). Therefore, the D509A mutant, unlike I703A altered several characteristics of the channel, thus the change in the capsaicin concentration response relationship could be because of secondary effects unrelated to PtdIns.

The L574A mutant was also predicted by our computational analysis to reduce PtdIns binding in the inhibitory site (Fig. 6*A*). This residue however may also interact with PtdIns(4,5)P_2_ in the activating binding site (Fig. 6*B*); therefore, its mutation may result in complex effects. Nevertheless, as the L574A mutation is predicted to have a large effect on the binding of PtdIns (Fig. 6*A*), we also tested the effect of this mutation on PtdIns inhibition. Application of 50 μM PtdIns induced a small and variable inhibition of the L574A mutant in the presence of 0.5 μM capsaicin and 50 μM PtdIns(4,5)P_2_ (Fig. S8), but this inhibition did not reach statistical significance.

Overall, our data with the I703A and L574A mutations give support to PtdIns inhibiting the channel *via* a binding site overlapping with that for vanilloid compounds such as capsaicin.

### Computational assessment of phosphoinositide specificity of binding sites in TRPV1

**Figure 8:**
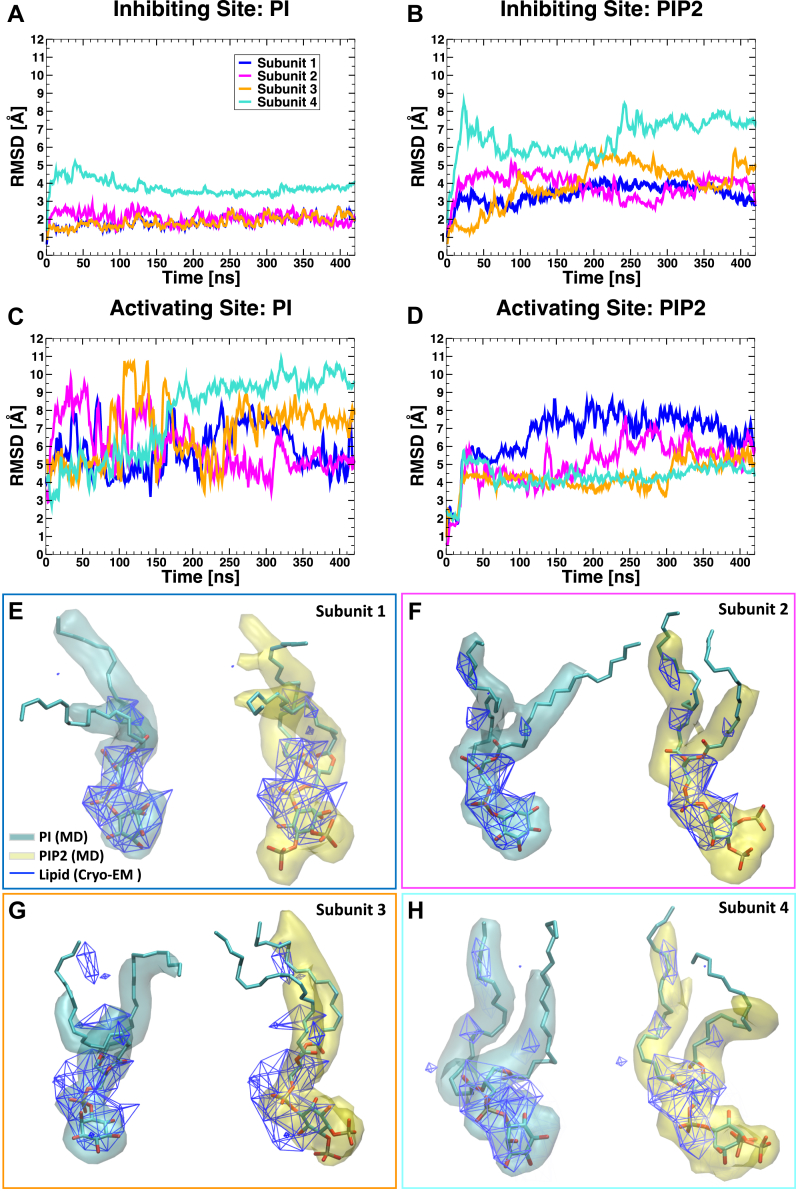
**Structural stability of PtdIns and PtdIns(4,5)P**_**2**_**at the inhibiting and the activating sites.***A–D*, root-mean-square deviation (RMSD) with respect to the initial configuration as a function of time from molecular dynamics simulations (a different color is used for each of the four symmetry-related subunits). *Panels A* and *B* show the RMSD of PtdIns (PI) and PtdIns(4,5)P_2_ (PIP_2_) respectively, when bound at the inhibiting site. *Panels C* and *D* show the RMSD of PtdIns and PtdIns(4,5)P_2_, respectively, when bound at the inhibiting site. *E–H*, superposition between the experimental and calculated lipid densities. Individual subunits are compared separately; each panel is color coded according to *panel A* legend. Calculated densities for PtdIns and PtdIns(4,5)P_2_ are colored in *cyan* and *yellow*, respectively, whereas the experimental electron density maps are shown as a blue-colored wireframe. PtdIns, phosphatidylinositol; PtdIns(4,5)P_2_, phosphatidylinositol 4,5-bisphosphate.

First, we assessed the binding site overlapping with the vanilloid site in the lipid nanodisc structure of TRPV1 (20). To this end, we performed MD simulations of TRPV1 with PtdIns or PtdIns(4,5)P_2_ in the putative inhibiting binding site. We compared the root-mean-square deviation (RMSD) of atomic positions of each phosphoinositide along the two MD trajectories (0.42 μs each, 3.36 μs cumulative over individual subunits). We found that PtdIns is more stable than PtdIns(4,5)P_2_ (Fig. 8, *A* and *B*, respectively), with RMSD values in the order of 1 to 3 Å for each individual subunit (with the exception of one reaching slightly higher RMSD values, up to 4 Å) for PtdIns *versus* RMSD values in the high 5 Å range (one subunit reaching up to 9 Å RMSD) for PtdIns(4,5)P_2_. The high structural stability and binding pose similarity of PtdIns at the inhibiting site is in strong agreement with our experimental evidence suggesting its role as a competitive vanilloid antagonist.

The lipid density in the vanilloid pocket of the TRPV1 structure was modeled as PtdIns, but it was argued that the site can accommodate other phosphoinositides, including PtdIns(4,5)P_2_ (20). Because the experimental structural model contains only a truncated version of the phospholipid tails and the stereochemistry of the inositol head group does not match the one naturally occurring in PtdIns, we calculated the electron density maps of PtdIns and PtdIns(4,5)P_2_ predicted by our MD simulations and compared them with the experimental lipid density map. As shown in Figure 8, *E*–*H*, PtdIns provides a much better fit between simulation and experimental density compared to PtdIns(4,5)P_2_.

Next, we performed a set of similar MD simulations with PtdIns or PtdIns(4,5)P_2_ bound at the putative activating site (two simulations, 0.42 μs each; 3.36 μs cumulative over all subunits). Analysis of RMSD values (Fig. 8, *C* and *D*) revealed average fluctuations higher than 4 Å for both phosphoinositide lipids, suggesting an overall larger mobility of both structure ensembles compared with their relative behavior at the inhibiting site (Fig. 8, *A* and *B*). This is not surprising, given the shallow nature of the activating site. The latter is located close to the pore, at the boundaries of the TM domain (Fig. 4, right inset), and partially faces the intracellular side of the membrane. Although detecting an overall enhanced mobility of both phosphoinositide lipids in the activating site, analysis of RMSD values (Fig. 8, *C* and *D*) also revealed significantly larger fluctuations for PtdIns (RMSD ranging between 3 Å and 11 Å; Fig. 8*C*) compared with PtdIns(4,5)P_2_ (RMSD ranging between 0.5 Å and 9 Å; Fig. 8*D*, a sign of greater binding instability for PtdIns at this site).

Overall our computational and experimental findings suggest that two phosphoinositide binding sites exist in the TRPV1 channel; one, the inhibiting site, is overlapping with the vanilloid pocket and binds primarily PtdIns. The other, the activating site, may favor binding of PtdIns(4,5)P_2_ over PtdIns.

## Discussion

### The inhibitory effect of PtdIns

The main goal of this work was to experimentally and computationally test the model in which phosphoinositides act as competitive antagonists binding to the vanilloid binding site (20), and to place this model into the context of the well-established positive effects of PtdIns(4,5)P_2_ and PtdIns(4)P. We found that PtdIns, but not PtdIns(4)P, partially inhibited TRPV1 in the presence of PtdIns(4,5)P_2_ in excised inside out patches. The inhibition by PtdIns showed an inverse correlation with the concentration of capsaicin, which is expected from a competitive inhibitor acting through a shared binding site.

We computationally identified residues in the vanilloid binding site that are predicted to interact with PtdIns, but not with capsaicin. We found that the D509A and I703A mutants shifted the capsaicin dose response to the left, as expected if PtdIns is a competitive antagonist of capsaicin binding. The mutant channels also became more sensitive to low pH, which is compatible with PtdIns not only acting as a competitive vanilloid antagonist but also as a negative allosteric regulator. This is in line with phosphoinositides also reducing heat sensitivity in lipid vesicles (31) as well as vanilloid antagonists, such as capsazepine and iodoresiniferatoxin, inhibiting TRPV1 responses to heat and low pH (30). The I703A mutation also eliminated inhibition by PtdIns in excised patches, but the D509A mutant was inhibited by PtdIns to similar extent to WT TRPV1. The D509A mutant however also reduced rectification and slowed deactivation kinetics of the channel; thus, it is possible that it altered capsaicin sensitivity because of secondary effects independent of PtdIns.

We also tested the functional effects of the L574A mutation, which was predicted to induce a large decrease in PtdIns affinity (Fig. 6*A*). This residue is also in contact with PtdIns(4,5)P_2_ in the activating binding site (Fig. 6*B*), which may induce complex effects when this residue is mutated. Consistent with this, the L574A mutation showed slightly right-shifted concentration dependence of capsaicin activation (Fig. S5*A*), which could be the consequence of allosteric effects by reduced PtdIns(4,5)P_2_ binding on capsaicin activation. Nevertheless, this mutant showed reduced inhibition by PtdIns (Fig. S8), consistent with its computationally predicated effect on PtdIns binding.

Further analysis of contact residues at the inhibiting site also revealed that 14 residues, namely R409, H410, D509, S510, Y511, S512, Y554, R557, E570, K571, I696, L699, Q700, and I703, established contacts with both PtdIns and PtdIns(4,5)P_2_ (Table S1). Interestingly, most of these residues have been also shown to prefer PtdIns over capsaicin (Fig. 5*A*), further supporting our finding that PtdIns competes with capsaicin for an overlapping, yet not identical, lipid binding site (*i.e.,* the inhibiting site *versus* the vanilloid binding site). Interestingly, two of these residues are negatively charged (D509 and E570) and exert important destabilization effects on the negatively charged head-group of PtdIns(4,5)P_2_ (Fig. S9*A*). Moreover, the additional steric bulk offered by the phosphate groups of PtdIns(4,5)P_2_ hardly fits within the inhibiting site, inducing the lipid head to interact with a cluster of peripheral residues (S402, S403, S404, and R499) located between the VSD and the TRP domain (Table S1 and Fig. S9*B*). While another residue, L574, was shown to clearly favor PtdIns over both PtdIns(4,5)P_2_ and capsaicin (Fig. 5*A*), the nearby R575 residue, which is located at the interface with the activating site, clearly prefers PtdIns(4,5)P_2_ (Fig. S9*B*). Although further work will be required to fully characterize the extent of PtdIns(4,5)P_2_ binding at the inhibiting site, our current data strongly support the hypothesis that at this site, PtdIns is more stable compared to PtdIns(4,5)P_2_.

While no negative effect of PtdIns(4,5)P_2_ was ever reported in excised patches, the purified TRPV1 incorporated into lipid vesicles showed reduced sensitivity to capsaicin when various phosphoinositides were incorporated at 4% (31). These experiments were performed in lipid vesicles containing ∼25% of the negatively charged phosphatidylglycerol, which likely satisfies the requirement of TRPV1 activity for a negatively charged lipid (15, 17). PtdIns and PtdIns(4)P induced a larger right shift in the capsaicin dose response than PtdIns(4,5)P_2_, and PtdIns(3,4,5)P_3_ had no effect (31). This order is the opposite of the effectiveness of phosphoinositides activating TRPV1 in excised patches where PtdIns(4,5)P_2_ and PtdIns(3,4,5)P_3_ had similar EC_50_ values (14), PtdIns(4)P had a significantly lower apparent affinity, but similar maximal effect (13, 14), whereas PtdIns had either no effect (13) or smaller than that induced by PtdIns(4,5)P_2_ (15) and current study.

The inhibitory effect of PtdIns(4,5)P_2_ in lipid vesicles may also be attributed to incorporation of this lipid into the extracellular leaflet of the membrane, as it was shown that application of either PtdIns(4,5)P_2_ or PtdIns(4)P to the extracellular membrane surface in outside out patches inhibited TRPV1 currents (32). This effect however is unlikely to be mediated by the vanilloid binding site, which is located in the intracellular portion of the transmembrane regions of TRPV1. Also, PtdIns had no effect when applied to outside out patches (32) but inhibited TRPV1 when incorporated symmetrically into lipid vesicles (31).

In addition to direct positive effects of PtdIns(4,5)P_2_, this lipid may exert indirect inhibitory effects on the channel *via* intermediary proteins (10) or modifying the effect of protein kinase C (9), but detailed discussion of these effects are beyond the scope of this manuscript.

### The activating effect of PtdIns

PtdIns(4,5)P_2_ is a well-established positive cofactor of TRPV1, and it was shown by several laboratories that it potentiates TRPV1 activity in excised patches (12, 13, 16). PtdIns(4)P was also shown to activate TRPV1 in excised patches, but it was less potent than PtdIns(4,5)P_2_ (13, 14). Here, we show that PtdIns also activates TRPV1 in the absence of PtdIns(4,5)P_2_, showing a more pronounced effect at higher capsaicin concentrations.

Thus, PtdIns exerts a dual effect on TRPV1; the inhibitory effect shows a negative correlation with capsaicin concentrations, whereas the positive effect shows a positive correlation with capsaicin concentrations. These data are not compatible with phosphoinositides and capsaicin acting on a single shared binding site. The simplest explanation is two binding sites, a site where PtdIns and PtdIns(4,5)P_2_ potentiates channel activity, and an inhibitory binding site, overlapping with the Vanilloid binding site where PtdIns inhibits channel activity.

The location of the PtdIns(4,5)P_2_ binding site in TRPV5 was recently determined experimentally (21). TRPV5 is constitutively active in a cellular environment, but its activity depends on the presence of PtdIns(4,5)P_2_, and unlike in the case of TRPV1, no inhibitory effect has been proposed for this channel. Consistent with this, the TRPV5 structure in the presence of PtdIns(4,5)P_2_ showed a conformational change indicating clear channel opening, when compared with the structure of the channel without this lipid.

The location of the PtdIns(4,5)P_2_ binding site responsible for opening TRPV5 is very similar to that computationally predicted to be responsible for activation in TRPV1 (16). Furthermore, we previously docked PtdIns(4,5)P_2_ to TRPV6 channels (21), and the location of the lipid showed remarkable similarity to the one experimentally determined for the closely related TRPV5 (21). By a similar approach, we used molecular docking to generate the starting configurations, for subsequent MD simulations, of PtdIns and PtdIns(4,5)P_2_ bound to TRPV1 in the inhibiting site that overlaps with vanilloid binding site and in the putative activating site. These configurations resulted to match almost perfectly with the experimental binding modes of lipids solved by cryo-EM, whenever available (20).

While the final proof for the location of the activating phosphoinositide binding site(s) in TRPV1 will likely come from future cryo-EM studies with added PtdIns(4,5)P_2_, our two binding site model is compatible with many seemingly controversial data in the literature.

Overall, we identify PtdIns as a negative regulator of TRPV1 activity by binding to a site that overlaps with the binding site for vanilloids. PtdIns bound to this site may serve to set the basal sensitivity of the channel. We propose a two phosphoinositide binding site model, where the distinct nonoverlapping activating binding site is mainly occupied by PtdIns(4,5)P_2_ in a cellular environment. Our data not only resolve a substantial controversy but also identify a novel, specific role for PtdIns in regulating ion channel activity.

## Experimental procedures

### *Xenopus laevis* oocyte preparation

Animal procedures were approved by the Institutional Animal Care and Use Committee at the New Jersey Medical School, and all animal procedures were performed in accordance with the approved guidelines. Oocytes were prepared from female *Xenopus laevis* frogs, as described earlier (33). Briefly, oocytes were digested using 0.2 mg/ml collagenase (Sigma) in a solution containing 82.5 mM NaCl, 2 mM KCl, 1 mM MgCl_2_, and 5 mM Hepes, pH 7.4 (OR2) overnight for ∼16 h at 18 °C in a temperature-controlled incubator. Defolliculated oocytes were selected and maintained in OR2 solution supplemented with 1.8 mM CaCl_2_ and 1% penicillin/streptomycin (Mediatech) at 18 °C.

### Excised inside-out patch clamp measurements in *Xenopus* oocytes

Excised inside-out patch clamp measurements were performed as described earlier (15). Briefly, cRNA of TRPV1 was transcribed from the rat TRPV1 clone in the pGEMSH vector (13) using the mMessage mMachine kit (Thermo Fisher). Point mutations were generated using the QuikChange XL Site-Directed Mutagenesis Kit (Agilent Technologies). cRNA was injected into *X. laevis* oocytes, and measurements were performed 5 to 7 days after injection, using borosilicate glass pipettes (World Precision Instruments) of 0.4 to 0.7 MΩ resistance, filled with a solution containing 96 mM NaCl, 2 mM KCl, 1 mM MgCl_2_, and 5 mM Hepes, (pH=7.4), supplemented with 0.5 μM or 4 μM capsaicin (as indicated in the figure legends). After establishing GΩ-resistance seals on devitellinized surfaces of oocytes, the inside-out configuration was established, and currents were measured using a ramp protocol from −100 to +100 mV applied every second. The main perfusion solution contained 96 mM KCl, 5 mM EGTA, and 10 mM Hepes, pH adjusted to 7.4. Currents were recorded with an Axopatch 200B unit and analyzed with the pClamp 9.2 software (Molecular Devices). Measurements were performed at 18 to 20 °C. Various stimulating solutions were applied to the internal side of the inside-out membrane patch using a custom-made gravity-driven perfusion system. DiC_8_ phosphoinositides were purchased from Cayman Chemical. Capsaicin was purchased from Sigma. Experiments were performed in a random order.

### Two-electrode voltage clamp in *Xenopus* oocytes

Measurements were performed as described earlier (13). Briefly, oocytes were placed in a solution containing 97 mM NaCl, 2 mM KCl, 1 mM MgCl_2_, and 5 mM Hepes, pH 7.4, and currents were recorded with thin-wall inner filament-containing glass pipettes (World Precision Instruments) filled with 3 M KCl in 1% agarose. Currents were measured with an GeneClamp 500B amplifier and analyzed with the pClamp 9.2 software (Molecular Devices) using the same ramp protocol as described earlier for excised patch measurements. Various stimulating solutions were applied using a gravity-driven perfusion system. Experiments were performed in a randomized order.

### Total internal reflection fluorescence microscopy

Human embryonic kidney (HEK-293) cells (ATCC catalogue number CRL-1573, RRID:CVCL_0045) were maintained in MEM (Life Technologies) with 10% FBS and 1% penicillin/streptomycin at 37 °C with 5% CO_2_. The cells were transiently transfected at 80% confluency with cDNA encoding the rat TRPV1 tagged with GFP on its N terminus (29) or with GFP-TRPV1-I703A, using the Effectene reagent (Qiagen). After 24 h, the transfected cells were plated on poly-L-lysine-coated 25-mm round coverslip (#1.5 thickness) (Fisher Scientific). The cells were used for TIRF imaging 2 days after transfection. Cells plated on the coverslip were placed into a recording chamber filled with extracellular solution containing (in mM) 137 NaCl, 5 KCl, 1 MgCl_2_, 10 Hepes, and 10 glucose (pH 7.4). TIRF images were obtained at room temperature using a Nikon Eclipse Ti2 microscope. Fluorescence excitation was performed using a 15-mW solid state 488-nm laser at 90% of the maximal power through a CFI Apochromat TIRF 60× oil objective (NA of 1.49), and the images were captured using an ORCA-Fusion Digital CMOS camera. The images were analyzed using Nikon NIS-Elements AR Analysis software and Image J.

### Data analysis

Data are represented as mean ± standard deviation plus scatter plots. Statistical significance was calculated either with *t* test (two tailed) or analysis of variance, with Bonferroni post hoc test for normally distributed data (Shapiro–Wilk test). For nonnormally distributed data, Wilcoxon or Mann–Whitney tests were used as appropriate. The specific tests for each experiment are described in the figure legends.

### Molecular docking and binding site mapping

We used the structure of TRPV1 in lipid nanodiscs solved in the presence of a phosphoinositide at the vanilloid binding site (apo TRPV1; PDB-ID: 5irz) (20) as the starting configuration for all our computational studies. Because the experimental structural model contains only a truncated version of the phospholipid tails and the stereochemistry of the sugar head group does not match any of the naturally occurring isomers of myo-inositol phosphate, we reconstructed, refined, and equilibrated the structure of the experimentally determined lipid. We used the charge and tautomeric states defined in (34). We thus docked the equilibrated structure of the refined lipid against the vanilloid binding site of TRPV1 (while preserving the experimental binding mode) before minimizing the obtained structural complex.

We used the docking tool Glide (35) (Schrödinger, LLC, 2018) to refine the binding mode generated manually for PtdIns against the inhibiting site of TRPV1. To this purpose, we started from the equilibrated structure of the channel (as described in the previous paragraph), which was properly prepared in the Maestro tool using the Protein Preparation Wizard (36). The docking grid was centered around the manually docked configuration of PtdIns. The structure of PtdIns was prepared using LigPrep. Docking poses were prioritized by score (kcal/mol), and postprocessing was performed by cluster analysis, using the tool g_cluster with the Jarvis Patrick methodology available in GROMACS (37, 38) (http://www.gromacs.org). The top four binding modes (one each TRPV1 subunit) were selected to constitute our final structural complex of TRPV1 with PtdIns bound at the inhibiting site. In addition, we modified the structures of PtdIns lipids in this final complex to match the geometry of PtdIns(4,5)P_2_ and by applying topology and atom types in agreement with the force field available through the CHARMM-GUI interface (SAPI25) (34). An analogous procedure was followed for the docking of PtdIns(4,5)P_2_ to the activating site.

### Molecular dynamics simulations

We embedded the structures of TRPV1 in complex with phosphoinositides in a hydrated 1-palmitoyl-2-oleoylphosphatidylcholine bilayer, using the membrane plugin of VMD (39). The system was then surrounded by 150 mM NaCl solution to reach an overall size of ∼160 × 160 × 150 Å^3^, with a total number of 330,000 atoms. We used all-atom MD simulation to equilibrate the systems (five in total: TRPV1 bound to PtdIns or PtdIns(4,5)P_2_ at the inhibiting or the activating sites plus a TRPV1 in complex with capsaicin) through a multistep protocol. First, we performed energy minimization of the systems (1000 steps). Second, we applied position restraints on protein (backbone and sidechain atoms) and lipids (head groups) that were gradually released during the first 50-ns simulation time (harmonic potentials with initial force constant K1 = 20 kcal/mol/Å^2^ were applied to all restrained atoms). Last, upon releasing the restraints, we performed additional ∼425-ns production runs. During all simulations, we used the velocity Verlet integration method to solve the equations of motion, with a time step of 2 fs using the Particle mesh Ewald method for calculating the electrostatic potential. We applied the Langevin temperature and Langevin piston coupling schemes. We set the temperature to 300 K and the pressure to 1 atm. We generated four independent MD trajectories, with PtdIns or PtdIns(4,5)P_2_ bound at the inhibiting or the activating sites. In all cases, we used the CHARMM36 force field (40) to describe the protein and the 1-palmitoyl-2-oleoylphosphatidylcholine lipids, and the parameters derived from ref (41). and ref. (34) for capsaicin and phosphoinositide lipids, respectively. The TIP3P model was used to describe water molecules (42). We used the VMD (39) (version 1.9) and NAMD (43) (version 2.12) programs for system preparation, equilibration, MD simulation, and trajectory analysis.

### Isolation of lipid densities (cryo-EM map)

We used the software Chimera (version 1.13.1; https://www.cgl.ucsf.edu/chimera/) to isolate densities of phosphoinositide lipids occupying the inhibiting site in the TRPV1 structure solved by cryo-EM (PDB-ID: 5irz). Specifically, the cryo-EM map was uploaded on the experimental structure file and the “Volume Viewer” and the “Volume Eraser” tools were used to extract the density blobs of bound lipids, which were saved as BRIX files. The experimental densities of lipids were then compared by superimposition with atomic densities derived from MD simulations.

### Atomic density maps calculations (MD simulations)

We calculated the atomic density maps for the phosphoinositide lipids (PtdIns or PtdIns(4,5)P_2_) occupying the inhibiting or the activating sites using the VolMap Plugin available in VMD(9) (version 1.9). For all calculations, we used a resolution of 0.5 Å, with an atom size of 1.0 Å and weights corresponding to the atomic mass. Maps were computed for all frames of each trajectory and subunit; frames were then combined by the averaging method. The computed atomic densities of lipids were then compared by superimposition with the experimental densities obtained by cryo-EM. Similarly, we generated the occupancy map of water molecules located within 6 Å of residue Q700 and capsaicin. The generated map was rendered as wireframe representation, colored in cyan (Fig. S4).

### Analyses of contact residues

We first used custom script (Tcl) to select protein sidechain atoms within a distance threshold (5 Å) of headgroups in either the phosphoinositide lipid PtdIns (PI) or capsaicin. Headgroups were defined as all heavy atoms in the substructures depicted in Figure 5*E* (bold licorice). Residue contact lists, with relative frequency of occurrence within the distance threshold, were then generated individually per subunit and finally combined over each simulation trajectory (TRPV1 with PtdIns or capsaicin at the inhibiting site). Last, relative frequencies were plotted as stacked bar charts, color-coded by ligand type (PtdIns is blue and capsaicin is gray), using Microsoft Excel. By comparing the two distributions, we identified eight protein sidechains that were selectively in contact with PtdIns (but not with capsaicin) in at least 50% of the analyzed simulation frames (dotted lines). These residues, namely D509, S510, K571, L574, I696, L699, Q700, and I703, are located in the inhibiting site of TRPV1.

### Residues in contact with PtdIns and PtdIns(4,5)P_2_ at the inhibiting site

In addition to computationally identify amino acid residues that are in contact with PtdIns, we identified residues interacting with PtdIns(4,5)P_2_ in the inhibiting site. We first calculated all contacts within a 5 Å cutoff established by PtdIns or PtdIns(4,5)P_2_. We then extracted all contact maintained for at least 50% of the frames in each simulation, by averaging over the four TRPV1 subunits. Results are reported in Table S1 and Figure S9, A and B, for PtdIns and PtdIns(4,5)P_2,_ respectively.

### Min-distance calculations

We calculated the minimum distance between the headgroups (Fig. 5*E*, bold licorice) of the phosphoinositide lipid PtdIns or capsaicin occupying the inhibiting site and the contact residues within 5 Å of PtdIns. Distances were calculated using the “g_mindist” function available with the program GROMACS (http://www.gromacs.org). Distance plots were generated in XMGRACE (https://www.linuxjournal.com/article/1218). Substructures were sketched using Sketch Tool available in Maestro (Schrödinger, LLC, 2018).

### ΔΔG calculations

We calculated the change in binding affinity of TRPV1 for PtdIns upon mutation of each PtdIns-contacting residue into alanine. To do so, we used the “Residue-Scanning and Mutation” protocol available in BioLuminate (44) (Schrödinger, LLC, 2018), as described in ref. (45). All residues (D509, S510, K571, L574, I696, L699, I703) were mutated to alanine sidechains; upon mutation, protein sidechains were minimized to optimize interactions with the bound lipid. Residue mutations and ΔΔ*G* calculations were performed on configurations sampled from the MD trajectory at ∼150-ns intervals. As a control, we repeated all calculations with capsaicin bound at the vanilloid site (one frame). Systems were prepared for the calculations using the Protein Preparation Wizard (36) (Schrödinger, LLC, 2018). For each system (four frames in total; three TRPV1 bound to PtdIns, and one TRPV1 bound to capsaicin), we performed independent calculations for each subunit and mutant (four subunits × eight mutants). We finally carried out a total of 32 calculations. Results were averaged over all subunits (Fig. 6). Affinity changes were plotted using Microsoft Excel.

## Data availability

All data are contained in the manuscript and in the supporting information. Molecular dynamics trajectories will be made available for academic use upon request to the authors.

## Supporting information

This article contains supporting information (20, 21).

## Conflict of interest

The authors declare that they have no conflicts of interest with the contents of this article.
