# Supplementary material for: Dual regulation of TRPV1 channels by phosphatidylinositol via functionally distinct binding sites
Source: J Biol Chem. 2021 Mar 23;296:100573. doi: 10.1016/j.jbc.2021.100573 (PMC8095115; doi:10.1016/j.jbc.2021.100573)
Supplement: Supplementary file 1 — Figures S1 to S9 and Table S1 [file mmc1.pdf]

Supporting information (Figures S1-S9, Table S1)

# Dual regulation of TRPV1 channels by phosphatidylinositol via functionally distinct binding sites

Aysenur Torun Yazici, Eleonora Gianti, Marina A. Kasimova, Bo-Hyun Lee, Vincenzo Carnevale and Tibor Rohacs

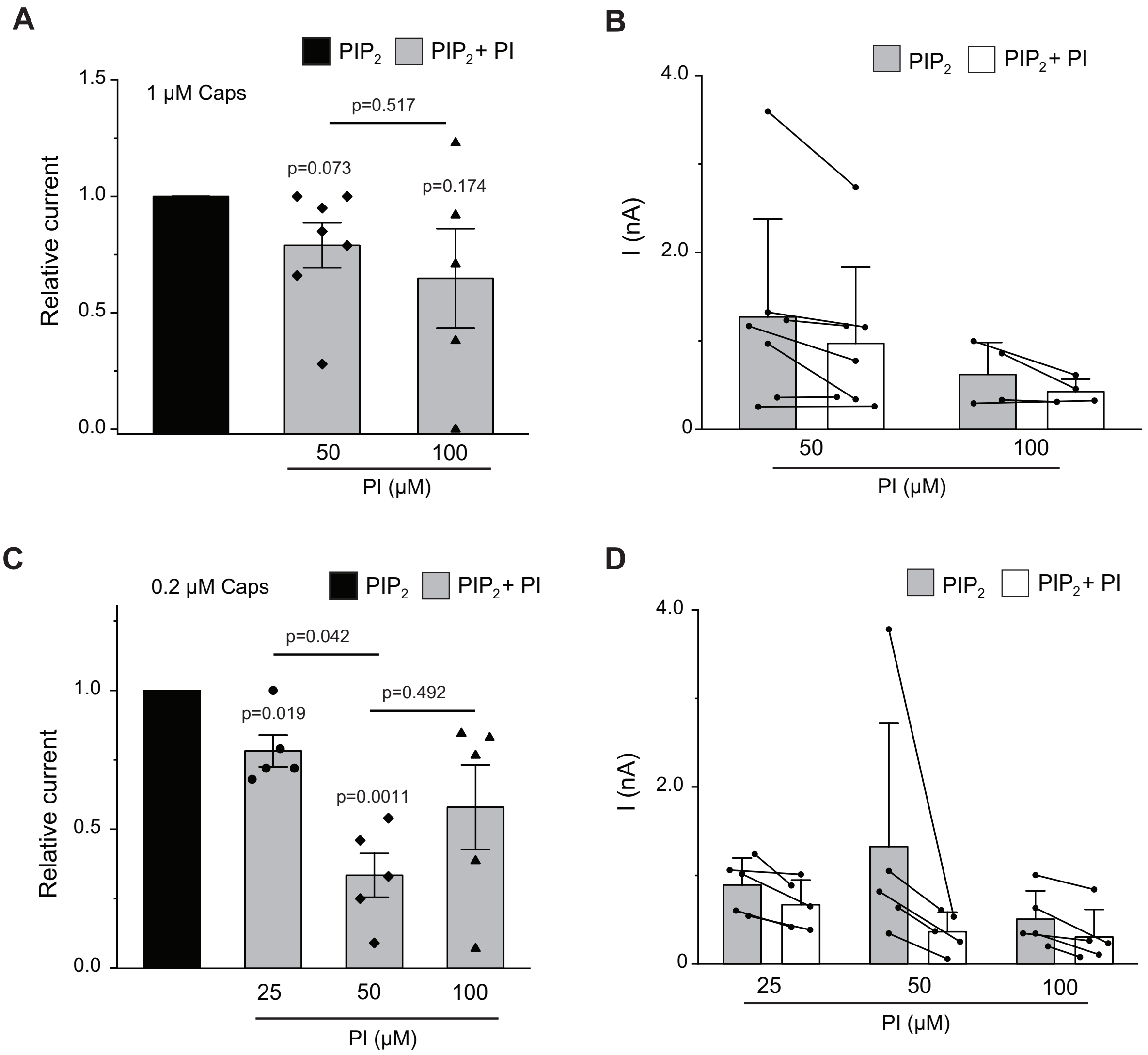


**<u>Figure S1.</u> Inhibition of TRPV1 currents by different concentrations of PtdIns**
Data summary from experiments similar to those shown in Figure 1A. **(A)** Current levels in the combined presence of 50 μM or 100 μM diC$_8$ PtdIns and 50 μM diC$_8$ PtdIns(4,5)P$_2$ normalized to the effect of 50 μM PtdIns(4,5)P$_2$ with 1 μM capsaicin in the patch pipette. **(B)** Raw current amplitudes evoked by PtdIns and PtdIns(4,5)P$_2$ from the same experiments. Data points from the same patches are connected. Data are shown as mean ± S.D. and scatter plots. (**C-D**) similar measurements with 0.2 μM capsaicin in the patch pipette. Statistical significance between the effects of PtdIns at different capsaicin concentrations was calculated with two sample t-test (A) or one-way ANOVA with Bonferroni post hoc test (C). P values above the individual columns show significance for PtdIns inhibition (difference from 1) calculated with one sample t-test.

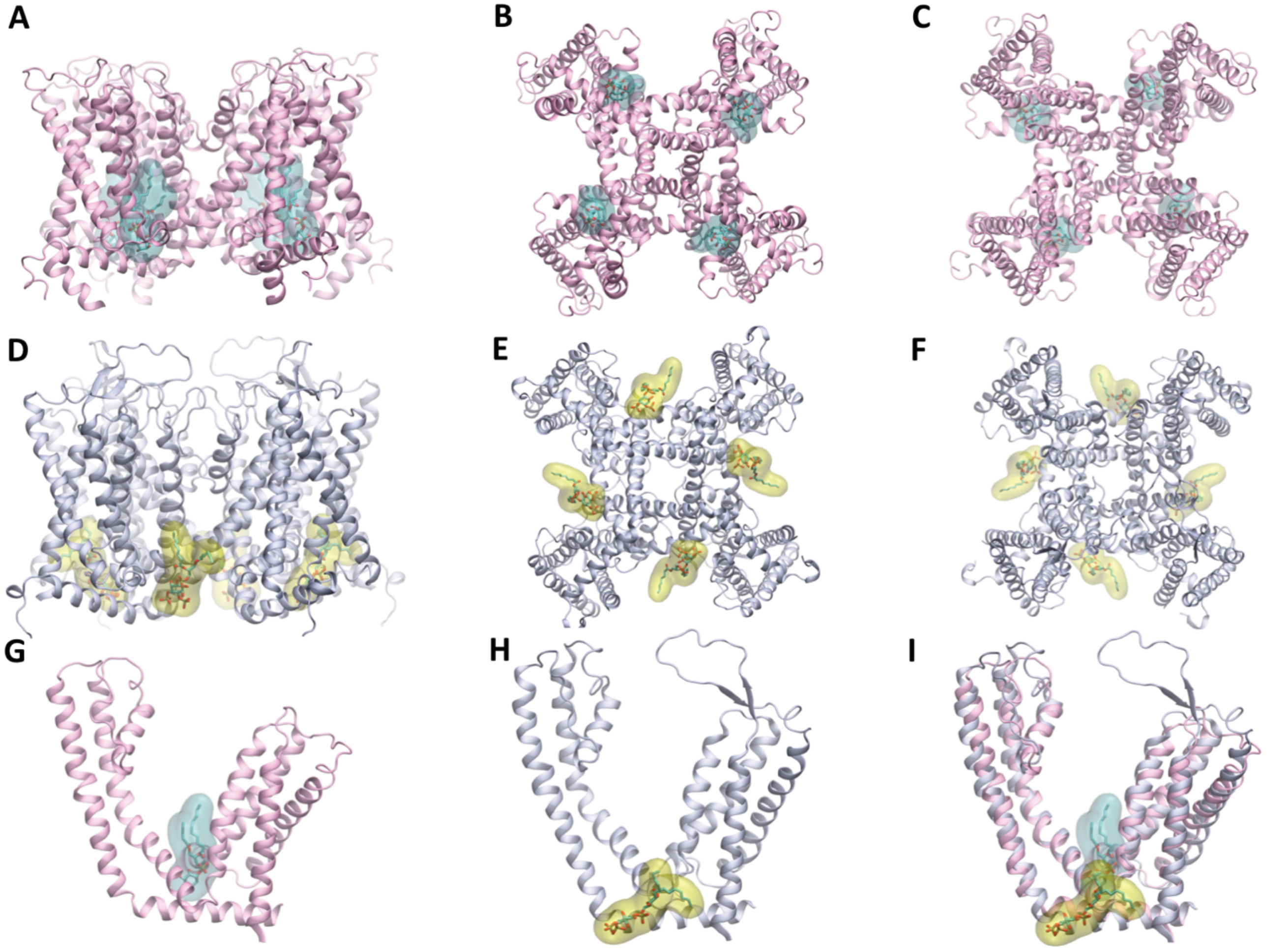


**Figure S2: Binding of PtdIns and PtdIns(4,5)$P_2$ at the inhibiting site and the putative activating site. (A-C).** PtdIns bound at the inhibiting site of TRPV1 (Gao et al., 2016); side, top and bottom views, respectively. **(D-F)**. PtdIns(4,5)$P_2$ bound at the activating site of TRPV5 (Hughes et al., 2018); side, top and bottom view, respectively**. (G).** PtdIns bound at the inhibiting site of TRPV1; only one subunit is shown. **(H).** PtdIns(4,5)$P_2$ bound at the activating site of TRPV5; only one subunit is shown. **(I)**. Superimposition between PtdIns and PtdIns(4,5)$P_2$ bound at the activating or inhibiting sites of TRPV1 or TRPV5, respectively. TRPV1 and TRPV5 are colored in pink and ice-blue, respectively. Phosphoinositide atoms are shown as balls and sticks, color coded by element: C, O and P atoms are grey, red and yellow, respectively; hydrogen atoms not shown. The molecular surfaces of PtdIns and PtdIns(4,5)$P_2$ are also shown in cyan and yellow for PtdIns and PtdIns(4,5)$P_2$, respectively.

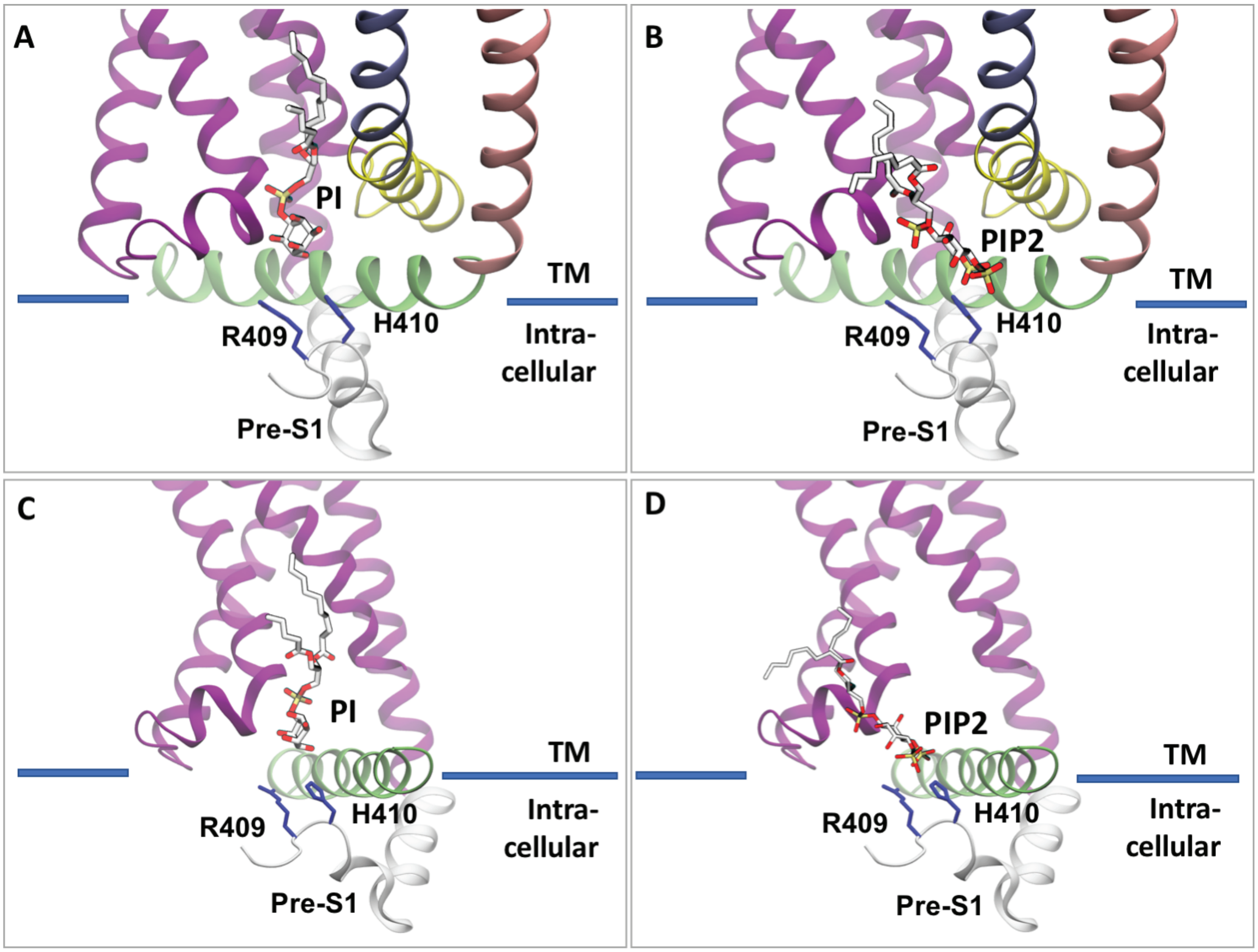


**<u>Figure S3:</u> Two charged amino acids selectively in contact with PtdIns are located on the pre-S1 segment.** Among the residues selectively in contact with PtdIns over capsaicin, two positively charged amino acids, namely R409 and H410, are located on the pre-S1 segment, too far away to engage in stabilizing interactions with PtdIns. Localization of R409 and H410 with respect to PtdIns and PtdIns(4,5)$P_2$ bound at the inhibiting **(A,C)** and the activating sites **(B,D),** respectively.

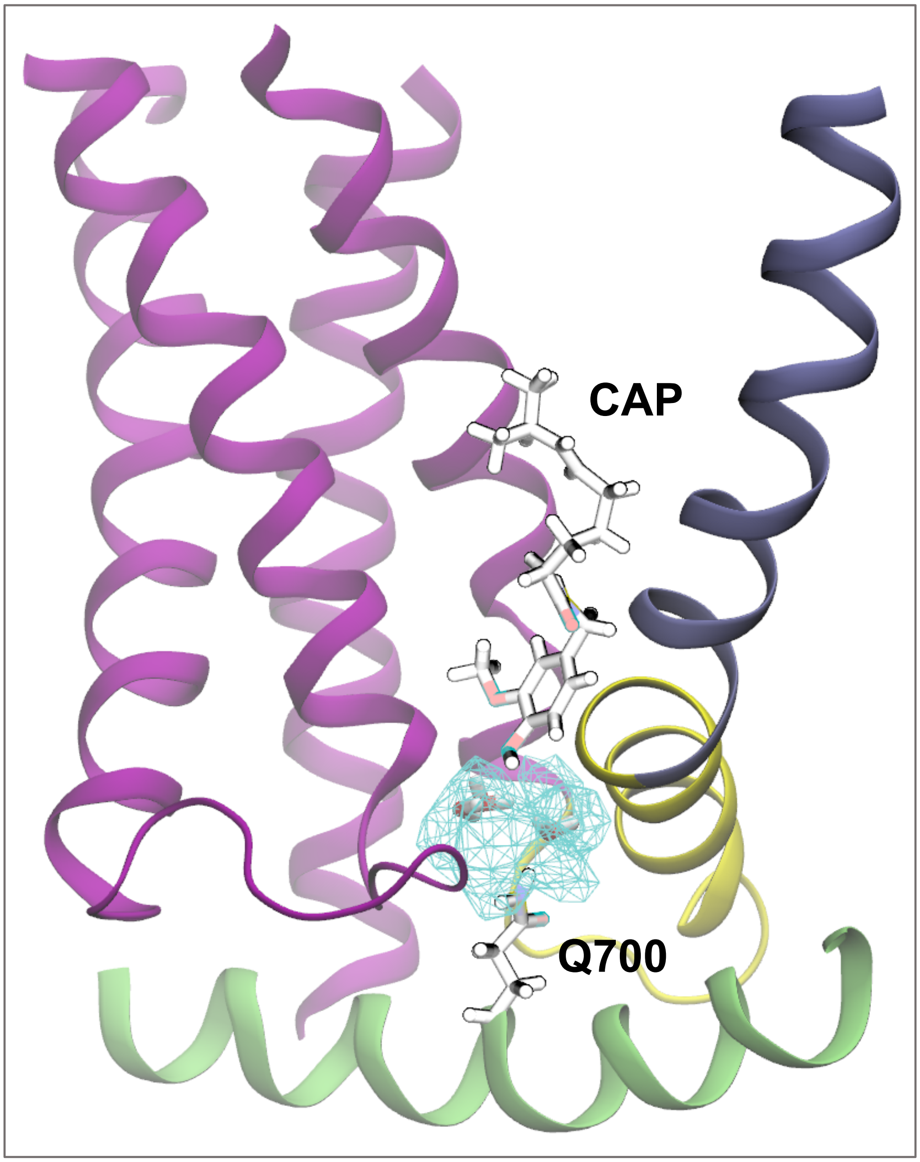


**<u>Figure S4</u>: Water mediated interaction between Q700 and capsaicin at the inhibiting site.** Sidechain of residue Q700 were observed in our molecular dynamics simulations to interact with capsaicin through an intervening hydrogen-bonded water molecule. Water occupancy maps calculated along the trajectory frames clearly indicate the localization of one or multiple water molecules in the proximity of both capsaicin and the side chain of residue Q700. Protein, water and capsaicin atom are rendered as in Figure 6B, colored in standard CPK rules. Water occupancy map rendered as wireframe, colored in cyan.

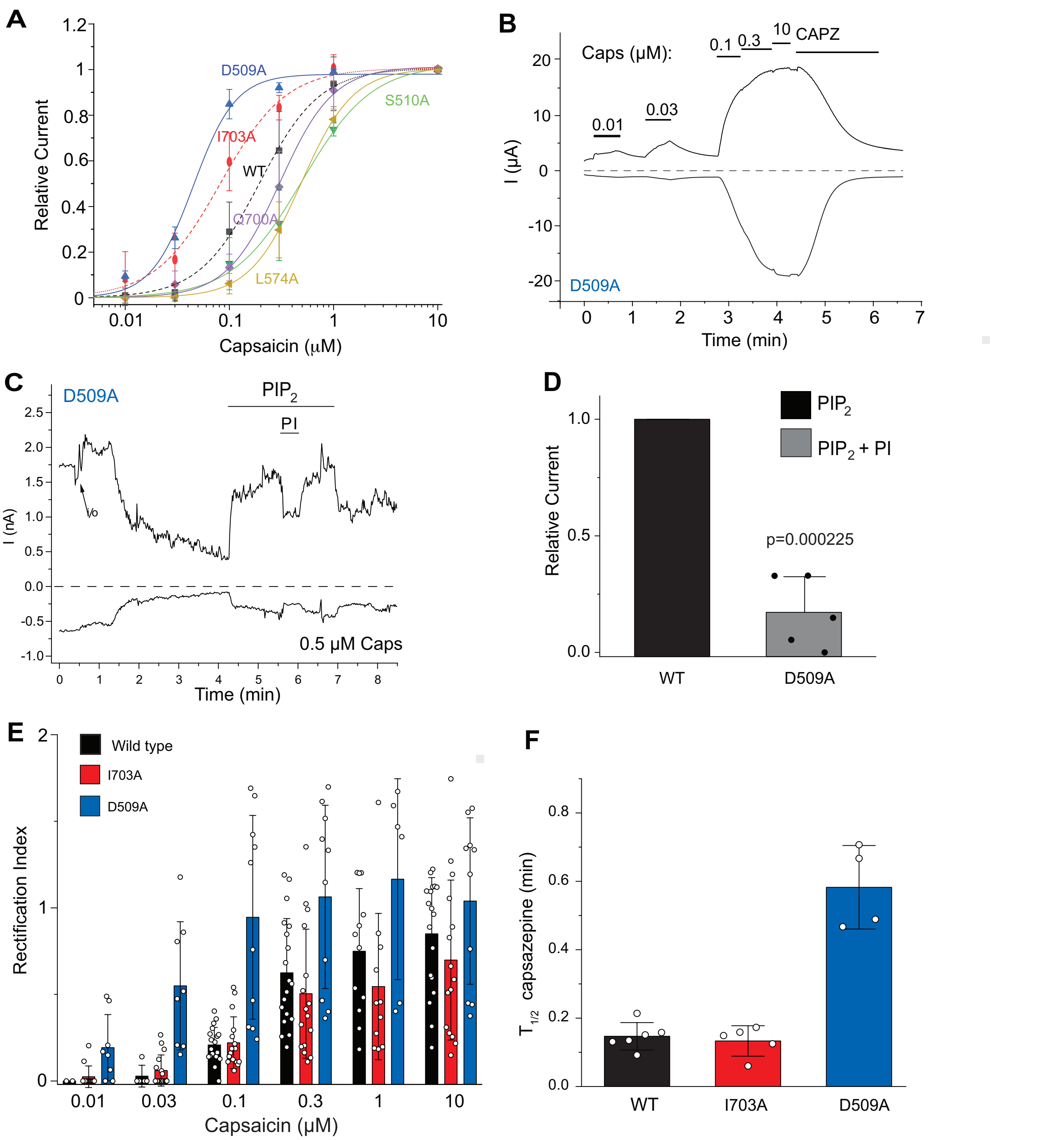


**Figure S5: The effects of additional TRPV1 mutations in the vanilloid binding site**. **(A)** Concentration response relationships of various mutants, wild type TRPV1 (WT) and I703A were replotted from Figure 7C. $EC_{50}$ values: D509A: 0.045 ± 0.004 μM; S510A: 0.500 ± 0.075; Q700A: 0.307 ± 0.033 μM; L574A: 0.490 ± 0.038 μM. **(B):** Representative two electrode voltage clamp trace for the D509A mutant, at -100 and +100 mV the applications of different concentrations of capsaicin (μM), and 50 μM capsazepine (CAPZ) are indicated by the horizontal lines. (**C**) Representative excised inside out patch clamp experiment for the D509A mutant, in the presence of 0.5 μM capsaicin in the patch pipette; the applications of 50 μM PtdIns(4,5)$P_2$ and 50 μM PtdIns are indicated by the horizontal lines. (**D**) Summary of the data; mean ± S.D. and scatter plots. Statistical significance (difference from 1) was calculated with one sample t-test. (**E**) Rectification index was calculated by dividing the current induced by various concentrations of capsaicin at -100 mV with that induced by the same concentration of capsaicin at 100 mV. Data are shown as mean ± S.D. and scatter plots. (**F**) $T_{1/2}$ of deactivation was calculated by measuring the time required to decrease to 50% of the current amplitude after the removal of 10 μM capsaicin and application of capsazepine from panel B and from Figure 7A,B. Data are shown as mean ± S.D. and scatter plots.

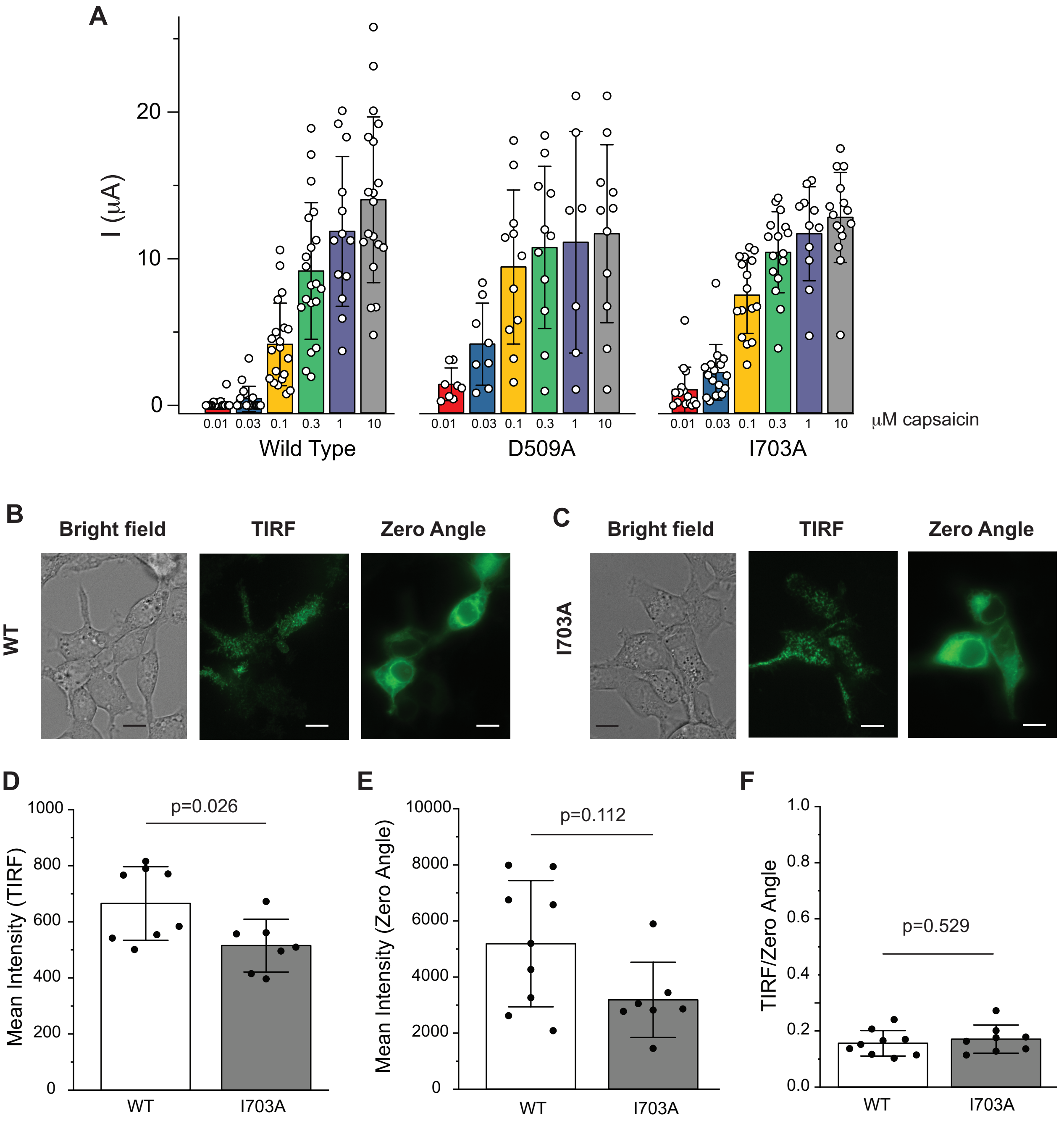


**<u>Figure S6.</u> Current amplitudes for the D509 and I703A mutant channels and surface expression levels for the I703 mutant. (A)** Current amplitudes evoked by different concentrations of capsaicin in two electrode voltage clamp experiments for wild type TRPV1, and for the D509A and the I703A mutants. Data are shown as mean ± S.D. and scatter plots. **(B-F)** total internal reflection fluorescence (TIRF) imaging experiments. Bright field, TIRF and wide field (zero angle) fluorescence representative images for wild type (B) and I703A (C). Scale bars show 10 µm. Data summary: TIRF intensity (D), mean zero angle intensity (E) and TIRF/zero angle (F). Data are plotted as mean ± S.D. and scatter plot. Individual points show average intensity of cells on one cover slip (5-27 cells). Pictures were taken from three randomly selected fields on each coverslip by an experimenter blind to the transfected construct. Data are shown from three independent transfections. Statistical significance was calculated with two sample t-test (D,F) and Mann-Whitney test (E).

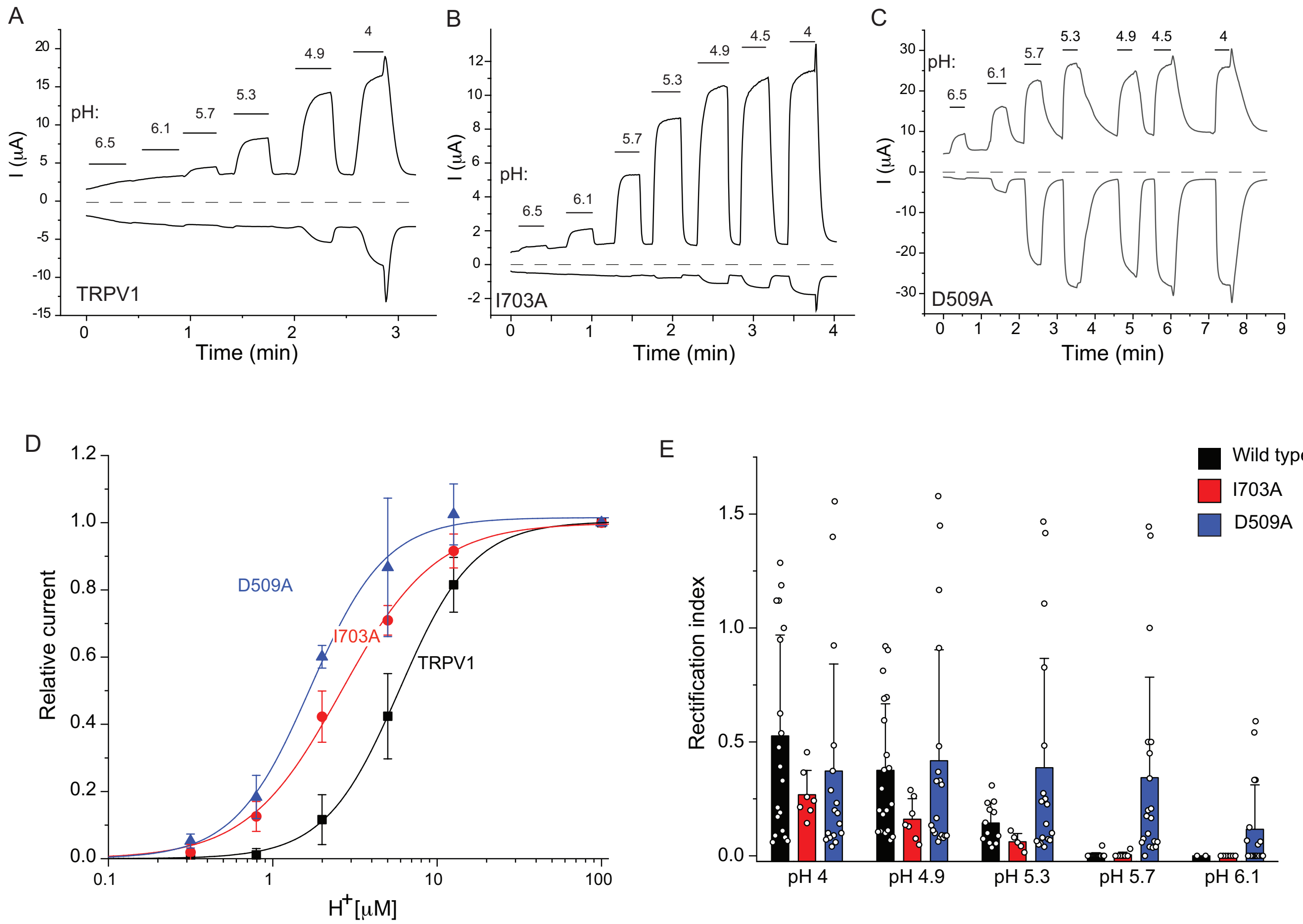


**Figure S7**: **The I703A and D509A mutants are more sensitive to activation by low pH**. Two electrode voltage clamp experiments were performed as described in the Methods section in Xenopus oocytes injected with the TRPV1 and mutant cRNA. **(A-C)** Representative current traces at -100 and +100 mV the applications of solutions with different pH are indicated by the horizontal lines. Dashed lines show zero current. **(D)** Hill plots for the sensitivity of activation by low pH at 100 mV, expressed as [$H^+$] in μM, symbols are mean ± S.D, n=5-7. $EC_{50}$ values: WT: 5.89 ± 0.36 μM (pK=5.23), I703A: 2.62 ± 0.12 μM (pK=5.58) and D509A: 1.69 ± 0.135 μM (pK=5.77). **(E)** Rectification index was calculated by dividing the current induced by various concentrations of capsaicin at -100 mV with that induced by the same concentration of capsaicin at 100 mV. Data are shown as mean ± S.D. and scatter plots.

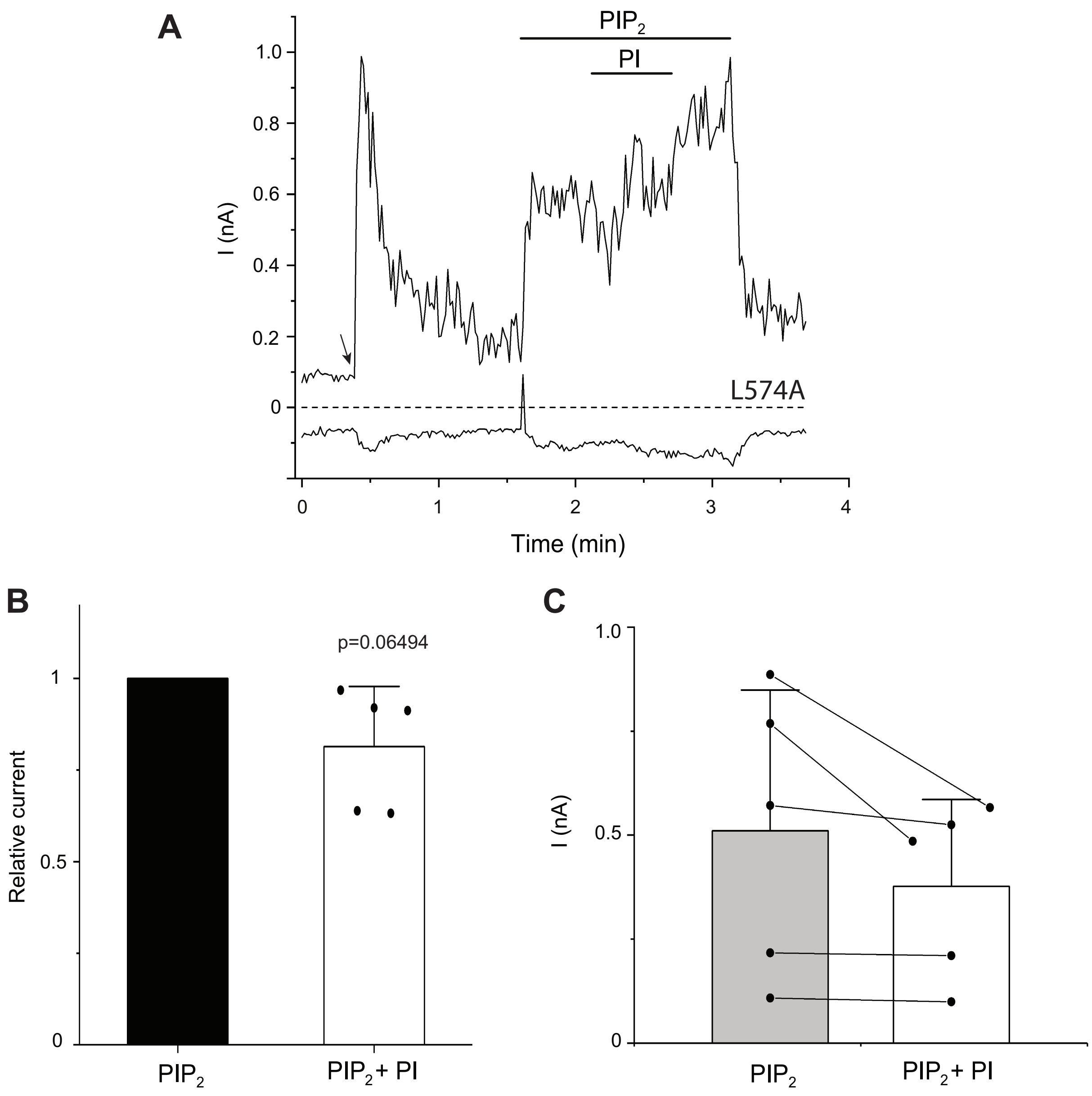


**<u>Figure S8.</u> Reduced PtdIns inhibition in the L574A mutant.** (A) Representative excised inside out patch clamp trace, the effects of 50 μM diC$_8$ PtdIns(4,5)P$_2$ and 50 μM diC$_8$ PtdIns are indicated by the horizontal lines. At the beginning of the measurement the excised patch was in a vesicle, which was broken by an air bubble at the time indicated by the arrow, establishing the inside out configuration. The patch pipette contained 0.5 μM capsaicin. (**B**) Summary data normalized to the current amplitudes evoked by PtdIns(4,5)P$_2$. Statistical significance (difference from 1) was calculated with one sample t-test. (C) Raw current amplitudes from the same measurements, current values obtained from the same patches are connected with straight lines. Data are shown as mean ± S.D. and scatter plots.

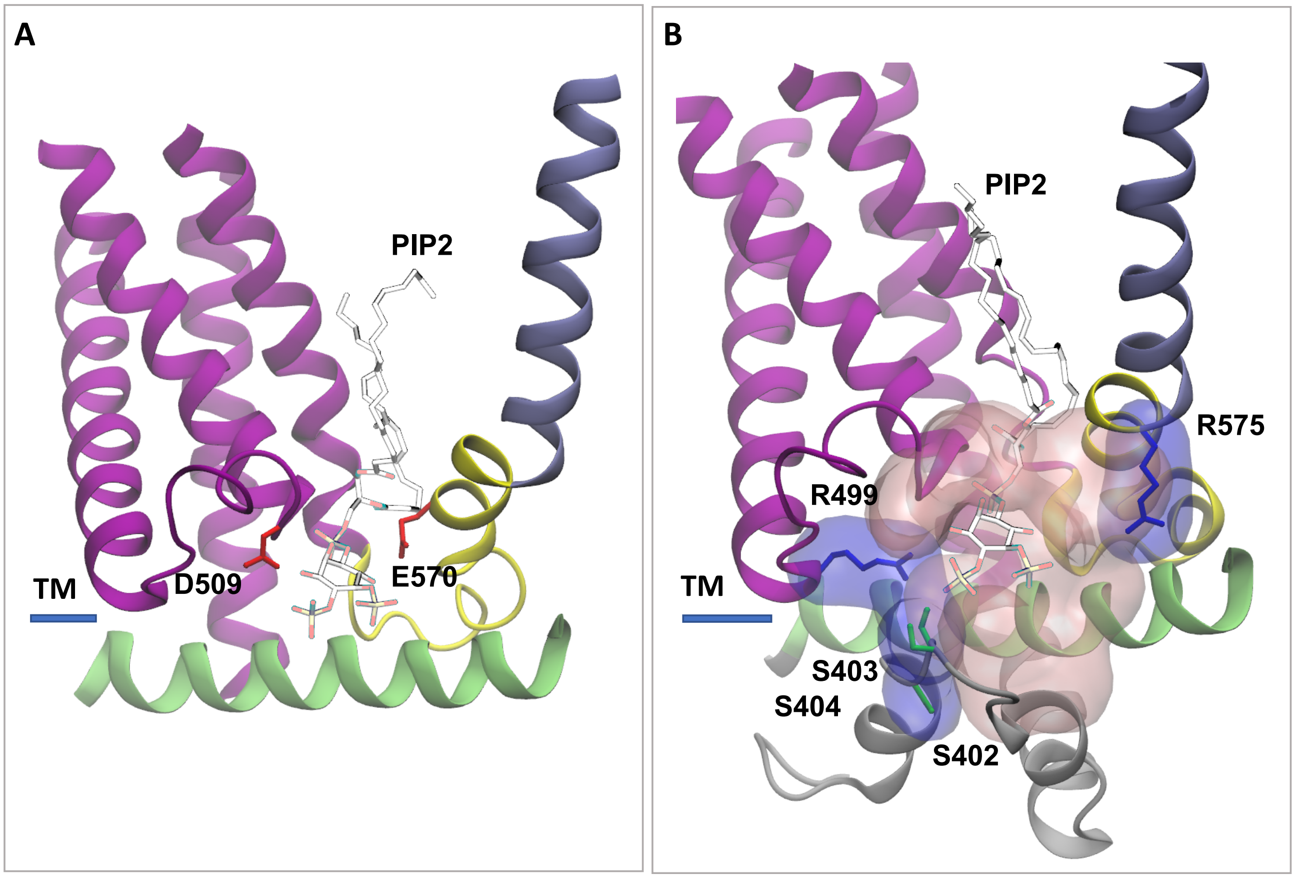


**<u>Figure S9</u>: Residues in contact with PtdIns(4,5)$P_2$ at the inhibiting site.** Analyses of residue contacts (from MD) identify what residues interact with the head groups of PtdIns(4,5)$P_2$ (Table S1). **(A)** Two negatively charged amino acid, D509 and E570, are in contact (within 5 Å) with the negatively charged head-group of PtdIns(4,5)$P_2$, thus providing destabilizing interactions. (**B**) Four residues (i.e. S402, S403, S404, R499) in contact with PtdIns(4,5)$P_2$ (Table S1) face a partially solvent exposed sub-pocket, which accommodates one of the lipid phosphate groups. The full list of interacting amino acids (contact residues) is provided in Table S1. In (**A**) & (**B**), protein backbone and lipid atoms are shown as in Figure 4. In **(A)**, negatively charged residues are shown in stick rendering, colored by residue type. In **(B)**, residues interacting with both PtdIns(4,5)$P_2$ and PtdIns are rendered as pink surface; residues interacting selectively with PtdIns(4,5)$P_2$ are rendered as blue surface. Selective contact residues are also shown as stick representation, colored by residue type. PtdIns(4,5)$P_2$ is also shown as stick representation, with standard CPK rules for atom coloring.

**Table S1: Residues interacting, either selectively or indiscriminately, with the head groups of PtdIns and PtdIns(4,5)$P_2$, at the inhibiting site.** Two negatively charged amino acids, D509 and E570 (in bold), provide destabilizing interactions with the negatively charged head-group of PtdIns(4,5)$P_2$.

| Amino-acid Residue | Interaction with PtdIns (% or Frames) | Interaction with PtdIns(4,5)$P_2$ (% of Frames) |
|---|---|---|
| R409 | 100 | 100 |
| H410 | 86.9 | 76.8 |
| **D509** | 100 | 99.7 |
| S510 | 97.1 | 95.6 |
| Y511 | 100 | 99.7 |
| S512 | 100 | 99.7 |
| Y554 | 100 | 99.6 |
| R557 | 100 | 99.7 |
| **E570** | 100 | 99.7 |
| K571 | 100 | 99.7 |
| I696 | 100 | 99.3 |
| L699 | 100 | 99.7 |
| Q700 | 100 | 99.7 |
| I703 | 100 | 99.0 |
| L574 | 95.6 | - |
| S402 | - | 65.1 |
| S403 | - | 74.8 |
| S404 | - | 81.2 |
| R499 | - | 100 |
| R575 | - | 99.7 |
